# Single‐Step Synthesis of Dithieno[3,2‐*b*:2′,3′‐*e*]Pyridines and Their Acid‐Dependent Optical and Photosensitising Properties

**DOI:** 10.1002/chem.71186

**Published:** 2026-05-27

**Authors:** Dominic Taylor, Matthew Wallace, Leonardo Amicosante, Gary S. Nichol, Scott J. Dalgarno, Filipe Vilela, Iain A. Wright, Neil B. McKeown

**Affiliations:** ^1^ EastCHEM School of Chemistry University of Edinburgh Edinburgh UK; ^2^ School of Engineering and Physical Sciences Heriot‐Watt University Edinburgh UK

**Keywords:** computational chemistry, dyes/pigments, photocatalysis, photophysics

## Abstract

Dithieno[3,2‐*b*:2‘,3’‐*e*]pyridine (**DTP**) is a heterocycle consisting of a central pyridine ring fused with two thiophene rings and can be regarded as a thiophene‐based analogue of the classic *N*‐heterocycle acridine. Unlike acridine‐based dyes, the fundamental chemistry of **DTP** has received relatively little attention. Here, we report the straightforward synthesis of a library of **DTP** molecules and an investigation of their electronic properties using UV/vis absorbance and emission spectroscopy supported by computational insights, with particular focus on the influence of protonation and methylation of the central N atom on their properties. While the absorbance of the **DTPs** studied was confined to ultraviolet wavelengths, protonation or *N*‐methylation of the pyridine group led to significant visible light absorption, which was exploited for the visible light photosensitisation of oxygen.

## Graphical abstract

The synthesis of dithieno[3,2‐*b*:2′,3′‐*e*]pyridines (**DTPs**) is herein reported, and their optical properties are studied both experimentally and computationally. Protonation or methylation of the nitrogen atom induced bathochromic shifts in their absorption and emission spectra. This allowed trifluoroacetic acid (TFA) to activate the photosensitising properties of the **DTPs** under visible light irradiation.

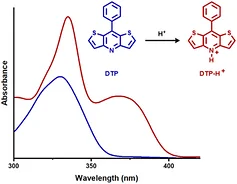

## 1 Introduction

**FIGURE 1:**
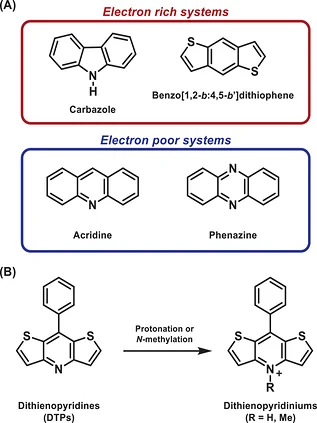
(A) Examples of common electron‐rich and electron‐poor heterocycles. (B) Chemical structure of **DTP** and its conversion into dithieno[3,2‐*b*:2′,3′‐*e*]pyridiniums *via* protonation or *N*‐methylation.

The nature and location of the heteroatom(s) in any given ring system have a commanding influence on the electronic structure of the system as a whole, notably their ability to donate or accept electrons. Electron donor heterocycles are electron‐rich systems such as carbazoles [1] and benzo[1,2‐*b*:4,5‐*b*′]dithiophenes [2, 3], while electron‐deficient systems include acridines [4] and phenazines (Figure 1A) [5]. The presence of the electronegative nitrogen atom(s) in these electron‐deficient heterocycles stabilises the *π*‐frontier molecular orbitals (FMOs), lowering their energy levels and thereby increasing the molecules’ electron‐accepting ability and tuning their optical and redox properties [6]. However, the lone pair of electrons on the nitrogen atom(s) of these molecules also renders them as Lewis bases, providing sites for protonation/*N*‐alkylation, with the resulting acridinium and phenazinium allowing for molecules to act as photobases [7].

Dithienopyridines consist of a central pyridine ring fused with two thiophene rings, and as such, they might be considered as thiophene analogues of the commonly encountered heterocycle acridine. There are three possible isomers of this heterocycle, but here we focus on dithieno[3,2‐*b*:2′,3′‐*e*]pyridine (herein abbreviated **DTP**), where the rings are fused such that the sulfur atoms are both *meta*‐ to the pyridine *N*‐atom (Figure 1B) [8]. While significant work has been conducted into acridine and its quaternerised derivatives as versatile photocatalysts [9], **DTPs** remain largely unexplored in the context of molecular materials, with existing reports focusing on their employment as building blocks in photovoltaic polymers [10, 11, 12], or in anion binding applications [13, 14].

Herein, we describe the synthesis of **DTP** derivatives featuring electron‐donating or withdrawing aryl and heteroaryl substituents on the *meso*‐position. We examine the relationship between their photophysical properties and chemical structure and have utilised protonation or *N*‐methylation of the pyridine nitrogen atom of these **DTPs** to exert control over the FMO energies and distributions. This induced significant bathochromic shifts in both the absorption and emission spectra of these dyes. While the absorption spectra of the neutral dyes were mainly limited to UV wavelengths, protonation/*N*‐methylation shifted the absorption into the visible region, which has also permitted some preliminary study of their potential for application as photosensitisers of singlet oxygen (^1^O_2_).

## 2 Results and Discussion

### 2.1 Synthesis and Photophysical Properties

Only a small number of reports detail the synthesis of **DTP**, with most utilising the acid‐catalyzed condensation of 3‐aminothiophenes with alkyl or aromatic aldehydes [8, 15, 16, 17]. An alternative method toward **DTPs** has been outlined by Oechsle et al. involving domino carbon‐sulfur cross‐coupling and 5‐*endo*‐dig cyclisation of alkynylated pyridines [18].

**SCHEME 1:**
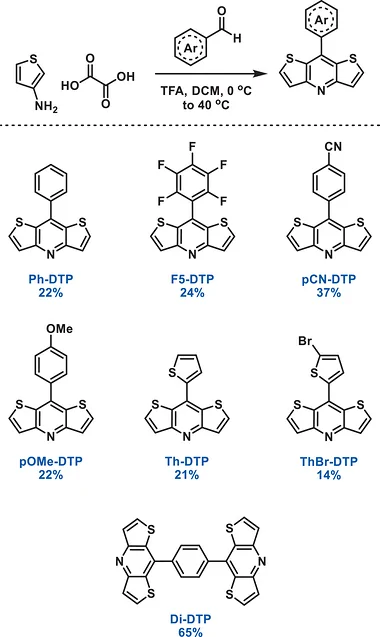
Synthesis of **DTPs** by TFA‐catalysed condensation of 3‐aminothiophene oxalate with aromatic aldehydes.

**SCHEME 2:**
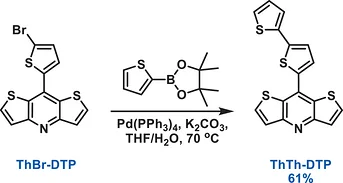
Synthesis of **ThTh‐DTP** from **ThBr‐DTP** by Suzuki–Miyaura cross‐coupling.

The first of these approaches was used toward the synthesis of the **DTPs** reported here using trifluoroacetic acid (TFA) for the acid‐catalysed condensation of 3‐aminothiophene oxalate with aromatic aldehydes to produce a library of *meso*‐substituted **DTP** derivatives (Scheme 1). This approach provided easy access to the **DTPs**, with purification achieved either by recrystallisation or simply washing the crude product with hot solvent. Isolated yields in the range of 14%–37% were obtained. To explore the limitations of this methodology, a further reaction was performed using terephthaldehyde, which produced the double pyridine decorated **Di‐DTP** in 65% yield. The structure of **Di‐DTP** is reminiscent of building blocks utilised in coordination polymers and metal–organic frameworks (MOFs) [19]. By utilising 5‐bromo‐2‐thiophenecarboxaldehyde, **ThBr‐DTP** could be obtained, which allowed for further functionalisation to be explored through cross‐coupling strategies. This was demonstrated by the Suzuki–Miyaura cross‐coupling of **ThBr‐DTP** with the pinacol ester of 2‐thiophene boronic acid to form **ThTh‐DTP** in 61% yield (Scheme 2).

**FIGURE 2:**
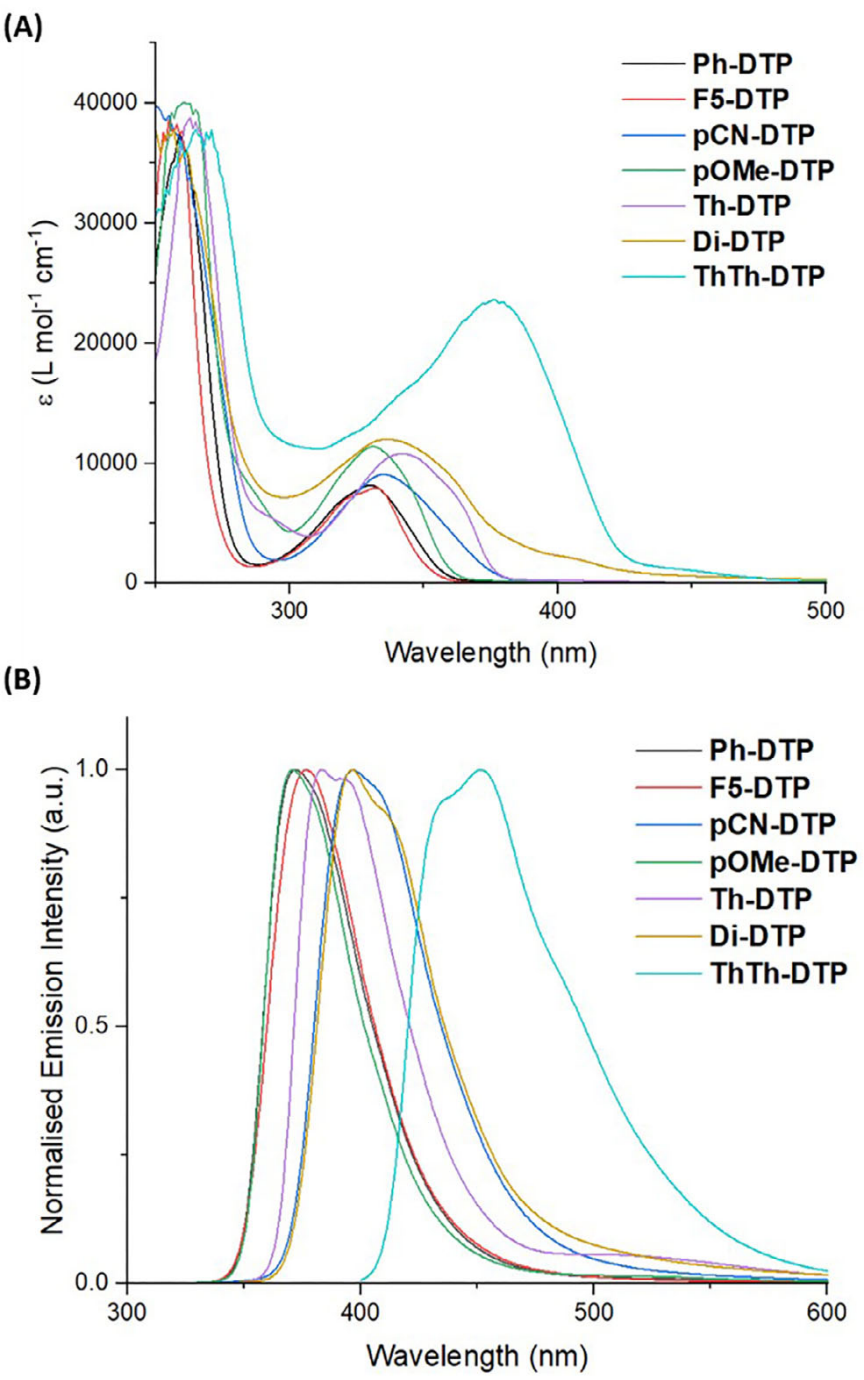
(A) UV–vis absorption and (B) normali**s**ed emission spectra of the **DTP** series in CHCl_3_ solution at a concentration of 100 µM.

UV/vis absorption and visible emission spectra were recorded in chloroform solution for each of the **DTPs** (Figure 2 and Table 1). In solution, the **DTPs** exhibited maxima of absorption (*λ*_abs_) in the UV region (330–377 nm) and emission maxima (*λ*_em_) falling in the range of 371–397 nm. The Stokes’ shifts for these compounds fell in the range of 3.22–4.66 × 10^3^ cm^−1^ and molar absorption coefficients (*ε*) were measured to be in the range of 8–24 × 10^3^ M^−1^ cm^−1^. The absolute photoluminescence quantum yield (PLQY) was measured for **Ph‐DTP** as 0.127.

**TABLE 1:** Photophysical properties of synthesised **DTPs** in CHCl_3_ solution.

Entry	Compound	*λ*_abs_ ^a^/nm	*λ*_em_ ^a^/nm	Stokes’ shifts/ × 10^3^ cm^−1^	*ε* ^a^, ^b^/ × 10^3^ M^−1^ cm^−1^
1	**Ph‐DTP**	330	372	3.42	8.24 ± 0.22
2	**F5‐DTP**	332	377	3.60	8.06 ± 0.09
3	**pCN‐DTP**	335	397	4.66	8.25 ± 0.01
4	**pOMe‐DTP**	331	371	3.26	10.0 ± 0.1
5	**Th‐DTP**	341	383	3.22	9.77 ± 0.08
6	**Di‐DTP**	336 (398^sh^)	397	4.57	12.1 ± 0.26
7	**ThTh‐DTP**	377 (440^sh^)	452	4.40	24.0 ± 0.76
8	**[Ph‐** **DTP**‐**Me** ^**+**^ **]** **[I^−^]**	374	—^c^	—	19.0 ± 1.0
9	**[Ph** **‐** **ACR** **‐** **Me** ^**+**^ **][I** ^**−**^ **]**	426	—^c^	—	18.2 ± 0.2

^a^ In CHCl_3_ solution. sh = shoulder.

^b^ The molar absorption coefficient (*ε*) was measured as described in Section 1 of the Supporting Information.

^c^ The sample was not emissive.

The absorption and emission spectra of **Ph‐DTP** were further examined across a range of solvents to assess its solvatochromic behaviour (Figure S16). The absorption maxima showed very little variation across the range of solvents tested, with all values falling in the range of 326–330 nm, whereas a much greater variation was observed in the range of emission maxima (359–90 nm). By plotting a Lippert–Mataga plot of Stokes’ shift versus the orientation polarizability function (Δ*f*), a positive correlation between the polarity of the solvent and the bathochromic shift in emission was observed. These results indicate that the lowest energy transition involves some degree of intramolecular charge transfer (ICT) (Figure S17 and Table S2) [20].

### 2.2 Effect of Protonation/*N*‐Methylation on DTPs

**FIGURE 3:**
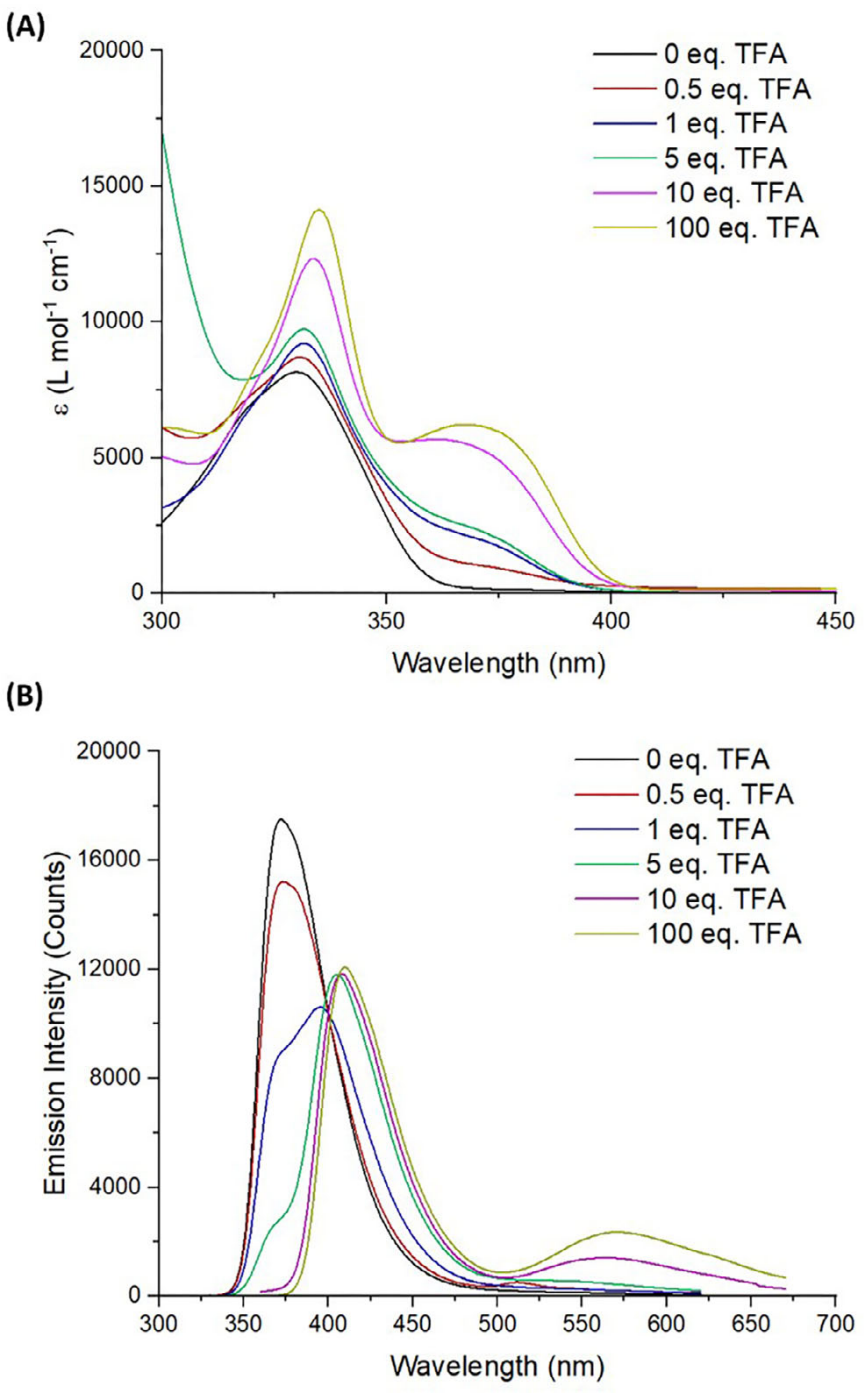
(A) UV–vis absorption spectra and (B) emission spectra of **Ph‐DTP** in CHCl_3_ solution at a concentration of 100 µM in the presence of 0–100 equivalents of TFA.

The pyridine nitrogen atom present within **DTPs** can be protonated or alkylated to form quaternary salts. TFA was employed as a Brønsted acid to examine the influence of protonation on the absorption and emission spectra. The addition of 0.5–100 equivalents of TFA to 100 µM solutions of **Ph‐DTP** in chloroform resulted in a hyperchromic increase of the main absorption peak and the gradual formation of a shoulder in the region of 350–410 nm (Figure 3). At 100 equivalents of TFA, this shoulder forms a well‐defined broad peak with a maximum located at 369 nm. In the fluorescence spectra, the addition of up to 100 equivalents of TFA results in the emission peak at 372 nm gradually decreasing with concomitant formation of a less intense peak at 410 nm. The formation of the protonated **Ph‐DTP** (**Ph‐DTP‐H** ^**+**^) was also observed upon the addition of 1 equivalent of TFA to a solution of **Ph‐DTP** in CDCl_3_. The peaks corresponding to the *α‐* and *β*‐protons on the thiophene groups underwent downfield shifts from 7.65 and 7.80 ppm to 8.06 and 8.13 ppm (Figure S1). These results are comparable to studies conducted by Hüßler et al. into the halochromic properties of dithienopyrazines: the addition of 0–100 equivalents of methanesulfonic acid also led to the formation of a lower energy peak in the absorption spectra and significant bathochromic shifts in emission [21]. Similar results have also been described by Rickertsen et al. when complexing acridine derivatives with Lewis acids such as Yb(OTf)_3_, Sc(OTf)_3_, or BF_3_.OEt_2_ [22].

The addition of TFA to solutions of the other **DTP** dyes in chloroform also resulted in the formation of a new shoulder in the absorption spectra at longer wavelengths than the parent **DTP**. For most of the **DTP** library, protonation moved the onset of light absorption to just within the blue region of the visible spectrum (Figures S19–S25) alongside bathochromic shifts in the emission spectra, with most of the new *λ*_em_ in the range of 410–457 nm. Protonation of the longest wavelength absorbing compound **ThTh‐DTP** (*λ*_max_ = 424 nm) facilitated absorption of light into the green region (Figure S25). The lowest energy emission maximum of **ThTh‐DTP** also shifted to 513 nm from an original value of 452 nm upon addition of 100 equivalents of TFA. Density functional theory (DFT) calculations performed on **Ph‐DTP** and **Ph‐DTP‐H^+^** revealed that protonation of the pyridine nitrogen atom decreased the energy difference (ΔE) between the HOMO and LUMO by *ca*. 0.5 eV, effectively causing the lowest energy absorption peak to bathochromically shift (Table S6). The experimentally observed drop in ΔE was 0.36 eV.

**FIGURE 4:**
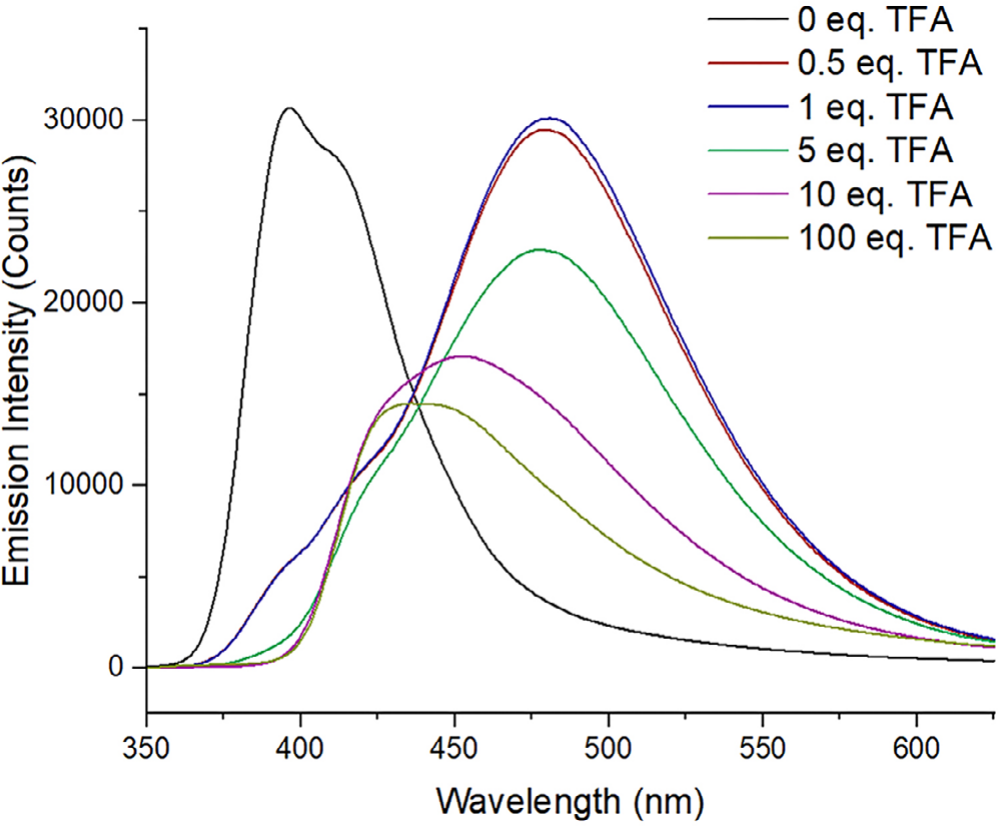
Emission spectra of **Di‐DTP** in CHCl_3_ solution at a concentration of 100 µM in the presence of 0–100 equivalents of TFA.

**FIGURE 5:**
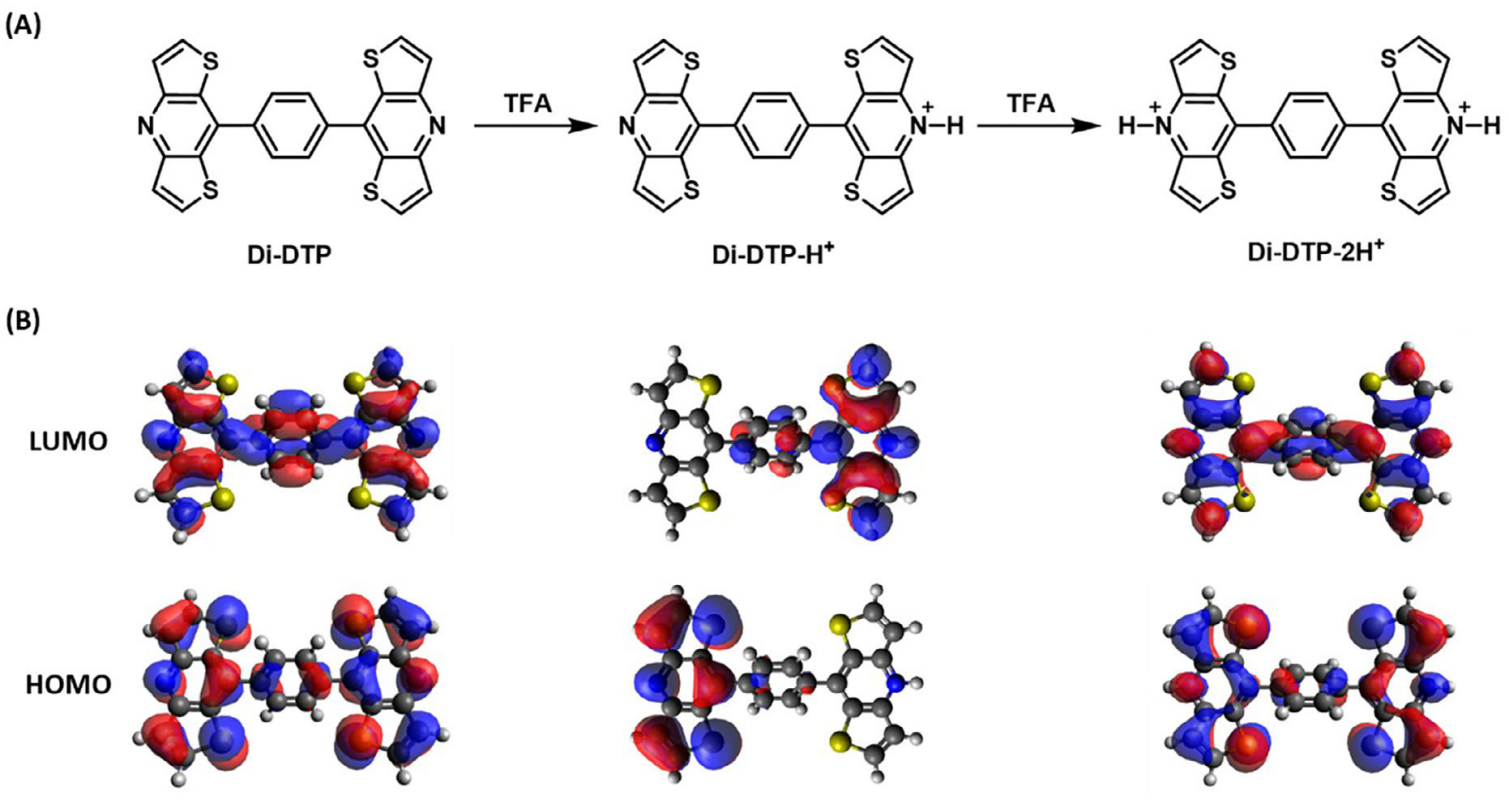
(A) Sequential protonation of **Di‐DTP**. (B) Calculated HOMO and LUMO of **Di‐DTP**, **Di‐DTP‐H** ^**+**^, and **Di‐DTP‐2H** ^**+**^.

In the case of **Di‐DTP**, a two‐step shift in the absorption and emission spectra was observed. The emission maxima bathochromically shifted from 397 nm to 480 nm upon the addition of 1 equivalent of TFA before hypsochromically shifting to 450 nm upon the addition of more than 5 equivalents of the acid (Figure 4). This can be explained by considering changes in the dipole moment of the molecule upon sequential protonation. The first protonation of **Di‐DTP** results in the dipolar donor–acceptor‐like structure **Di‐DTP‐H** ^**+**^, leading to the bathochromic shift in emission (Figure 5A). The second protonation to form **Di‐DTP‐2H** ^**+**^ then reduces the dipolar character, and a hypsochromic shift is observed. The symmetric band shape of the **Di‐DTP** **‐** **H** ^**+**^ is suggestive of greater charge transfer character compared to the ground and doubly‐protonated form [23]. These assignments are further supported by changes in the FMO distributions of **Di‐DTP**, **Di‐DTP‐H** ^**+**^, and **Di‐DTP‐2H** ^**+**^ (Figure 5B). The neutral state has a uniform distribution of both the HOMO and the LUMO. Upon protonation, the HOMO and LUMO are now located over the neutral **DTP** and the positively charged **DTP‐** **H** ^**+**^ rings, respectively, while the doubly‐protonated form re‐assumes a uniform distribution of both HOMO and LUMO.

**SCHEME 3:**
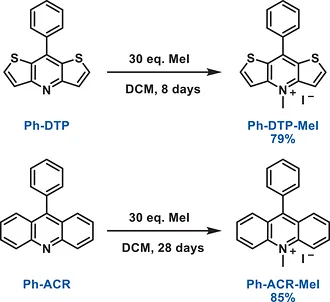
*N*‐Methylation of **Ph‐DTP** and 9‐phenylacridine using methyl iodide.

**FIGURE 6:**
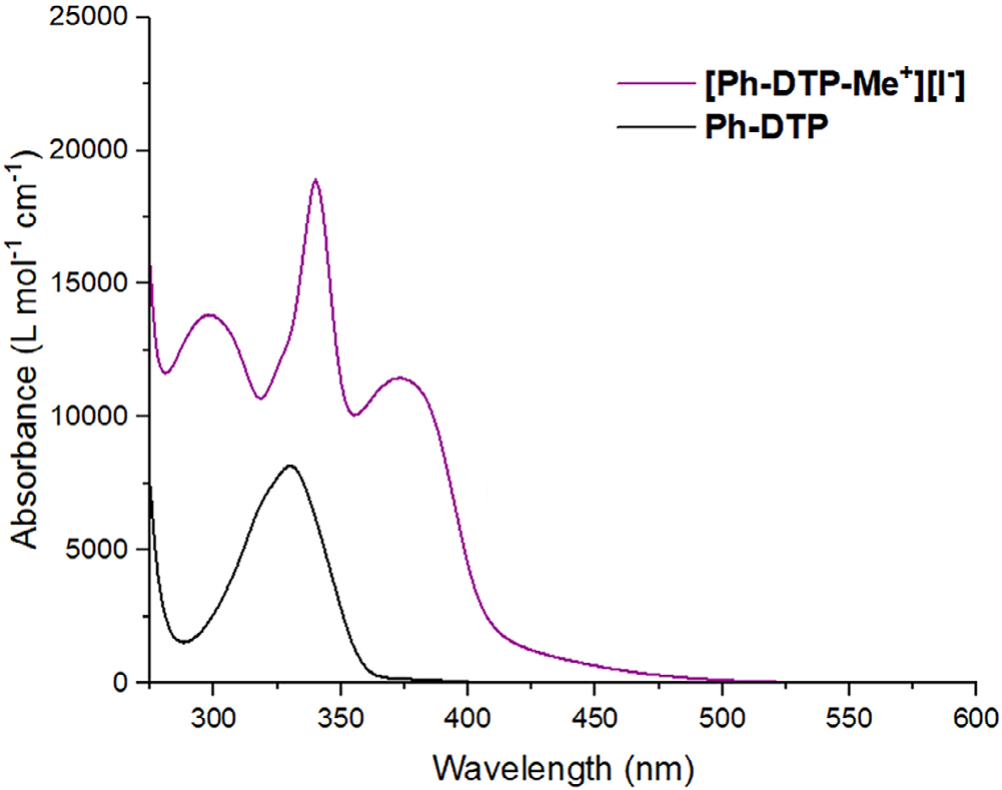
UV–vis absorption spectra of **Ph‐DTP** and **[Ph** **‐** **DTP** **‐** **Me** ^**+**^ **][I** ^**−**^ **]** in CHCl_3_ solution at a concentration of 100 µM.

In addition to testing the effect that protonation had on the absorption and emission of **Ph‐DTP**, the effect of *N*‐methylation at the pyridine group of **Ph‐DTP** was also investigated by preparing **[Ph‐DTP‐Me** ^**+**^ **]** **[** **I** ^**−**^ **]** using methyl iodide (Scheme 3). The reaction proceeded extremely slowly, requiring 8 days to achieve full conversion even in the presence of 30 equivalents of methyl iodide, yielding **[Ph‐DTP‐M** **e** ^**+**^ **]** **[** **I** ^**−**^ **]** in 79% yield. The process of *N*‐methylation of **Ph‐DTP** was accompanied by a significant downfield shift (ca. 0.7 ppm) in the peaks of the *α*,*β*‐thiophene protons (Figure S2): this change was similar to the shift observed upon protonation of **Ph‐DTP** using TFA (Figure S1). To provide a comparison for **Ph‐DTP**, *N*‐methylation of 9‐phenylacridine was also performed using the same synthetic conditions, with 85% yield of the target product obtained after 28 days. Compared to **Ph‐DTP**, **[Ph‐DTP‐M** **e** ^**+**^ **]** **[** **I** ^**−**^ **]** in chloroform solution exhibited stronger absorption of light at 341 nm as well as the emergence of a broad peak with a local maximum at 374 nm with an onset of absorption at 409 nm (Figure 6). Unlike **Ph‐DTP** in the presence of TFA, **[Ph‐DTP‐M** **e** ^**+**^ **]** **[**I^**−**^ **]** did not exhibit any fluorescence under UV‐illumination in either chloroform solution or the solid state.

### 2.3 DFT Modeling of DTPs

To complement the experimental characterisation of the library of **DTP** molecules, DFT calculations were conducted at the B3LYP/6‐31g++(d,2p) level. This included calculating the energies of the HOMO and LUMO as shown in Table S5. For neutral **DTPs**, the HOMO was primarily localised on the **DTP** moiety, whilst the LUMO has a greater density on the aryl group. This indicates there is a degree of charge transfer character in the lowest energy absorption and agrees with the solvatochromism study. The addition of a second thiophene group to **Th‐DTP** to yield **ThTh‐DTP** changes the structure of the HOMO and LUMO, with both FMOs featuring significant contributions from the 2,2’‐bithiophene group. This would suggest that the lowest energy transition for **ThTh‐DTP** is in fact the *π*–π* transition.

**FIGURE 7:**
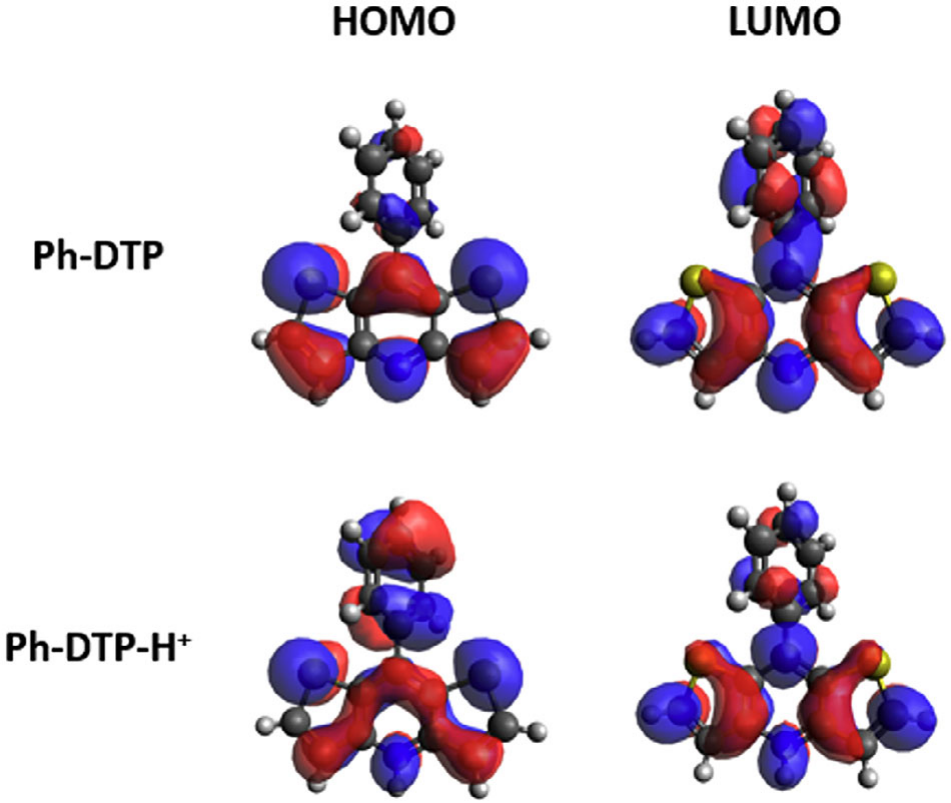
HOMO and LUMO of **Ph‐DTP** and **Ph‐DTP‐H** ^**+**^ calculated using DFT at the B3LYP/6‐31g++(d,2p) level.

Protonation or methylation of the **Ph‐DTP** at the pyridine nitrogen atom was also modelled by DFT methods, with the FMOs of **Ph‐DTP‐H** ^**+**^ and **Ph‐DTP‐M** **e** ^**+**^ showing almost the opposite behavior to that observed for the neutral **DTP** molecules (Table S6). Following protonation, the HOMO of **Ph‐DTP‐** **H** ^**+**^ now shows additional significant contributions from the *meso*‐substituent while the LUMO is largely located on the **DTP** system (Figure 7). *N*‐Protonation/methylation of **Ph‐DTP** was accompanied by a significant reduction in the energies of both FMOs: the net result was a reduction in the HOMO–LUMO gap (Δ*E*) by ca. 0.5 eV for both **Ph‐DTP‐** **H** ^**+**^ and **Ph‐DTP‐M** **e** ^**+**^ relative to **Ph‐DTP**.

In the case of **Di‐DTP**, the ability of the molecule to protonate either once or twice leads to significant changes in the distribution of the HOMO and LUMO (Figure 5B). In its neutral form, the lowest energy transition of **Di‐DTP** is a *π*–π* transition with the LUMO presenting significant quinoidal character. Protonation of one **DTP** group (**Di‐DTP‐H** ^**+**^) changes the nature of the lowest energy transition to ICT: the protonated **DTP** group acts as the electron acceptor group while the non‐protonated **DTP** group acts as the electron donor. The doubly protonated **Di‐DTP‐2** **H** ^**+**^ is akin to **Di‐DTP**, with the lowest energy transition reverting to *π*–π* with quinoidal character in the LUMO.

### 2.4 Single Crystal X‐Ray Structures

**FIGURE 8:**
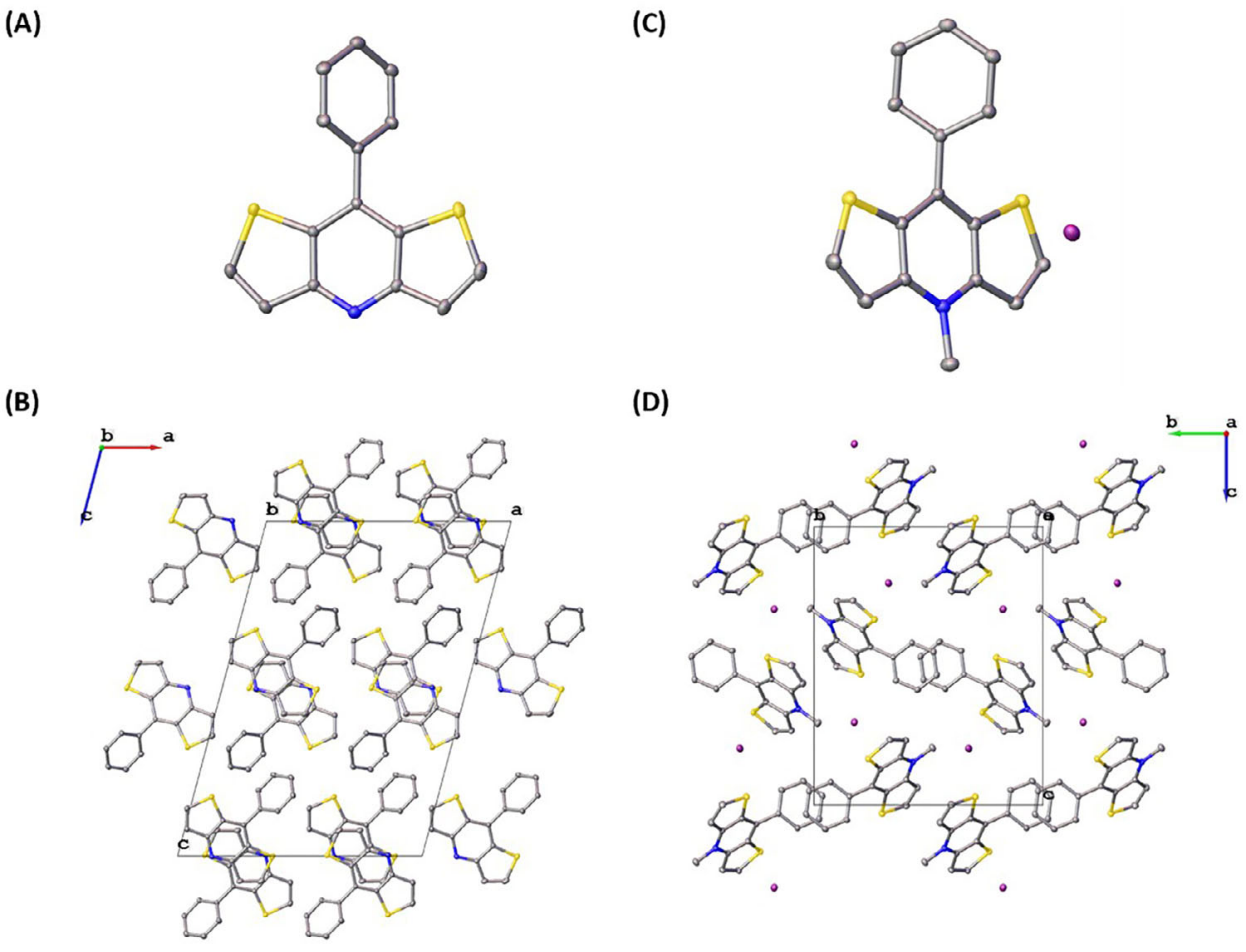
Single crystal X‐ray structures of (A) **Ph‐DTP** and (B) **[Ph‐DTP‐M** **e** ^**+**^ **]** **[** **I** ^**−**^ **]**. Packing diagrams for (C) **Ph‐DTP** as viewed down the *b*‐axis and (D) **[Ph‐DTP‐M** **e** ^**+**^ **][** **I** ^**−**^ **]** as viewed down the *a*‐axis. The atoms are shown as thermal ellipsoids at 50% probability. The hydrogen atoms have been omitted for clarity.

Single crystal X‐ray structures for **Ph‐DTP**, **F5‐DTP**, **pOMe‐DTP**, and **Th‐DTP** were determined and are presented in the Supporting Information (Figures S4–S7) and stored in the Cambridge Structural Database (CCDC deposition numbers 2503850–2503853) [24, 25]. In the case of **Ph‐DTP**, the crystal structure exhibited *π*–*π* interactions between the **DTPs** and C─H…*π* interactions between the *meso*‐phenyl groups (Figure 8A,C). The single crystal X‐ray structure of **[Ph‐DTP‐M** **e** ^**+**^ **]** **[** **I** ^**−**^ **]** was also obtained (CCDC deposition number 2503854) (Figure S8), confirming the presence of the methyl group and iodine counterion (Figure 8B). In contrast to **Ph‐DTP**, the crystal structure of **[Ph‐DTP‐M** **e** ^**+**^ **]** **[** **I** ^**−**^ **]** exhibited *π*–*π* stacking of the *meso*‐phenyl groups (Figure 8D). Notably, the structure of **[Ph‐DTP‐M** **e** ^**+**^ **]** **[** **I** ^**−**^ **]** also deviated slightly from planar to accommodate the iodide anion. C─H…iodine interactions were observed between the iodine anion and the **DTP**, *N*‐methyl, and *meso*‐phenyl groups.

### 2.5 Photosensitising Properties of DTPs

**SCHEME 4:**
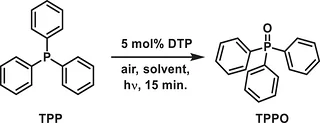
Photooxidation of TPP to TPPO using **DTP** photosensitizers.

Due to the onset of significant visible light absorption brought about by either protonation or *N*‐methylation of the **DTPs**, it was envisaged to test their ability to generate singlet oxygen (^1^O_2_) under exposure to violet (420 nm) light. This was investigated using the oxidation of triphenylphosphine (TPP) to triphenylphosphine oxide (TPPO) as the test reaction (Scheme 4): TPP is commonly used as a trap molecule for ^1^O_2,_ as the production of TPPO can be easily monitored by quantitative ^31^P NMR spectroscopy, and the reaction does not result in by‐products (Figure S30) [26]. The initial set of reaction conditions tested the oxidation of TPP (0.2 mmol) using **[Ph** **‐** **DTP** **‐** **Me** ^**+**^ **][I** ^**−**^ **]** at 5 mol% loading as the photosensitiser in various solvents. After 15 min, up to 87% conversion of TPP to TPPO was achieved using CDCl_3_ as the solvent (Table S3, entry 1), suggesting that **[Ph** **‐** **DTP** **‐** **Me** ^**+**^ **][** **I** ^**−**^ **]** was indeed generating ^1^O_2_. Other solvents were tested in this reaction, including CHCl_3_, DCM, acetonitrile, and DMF, returning conversions of 85%, 44%, 85%, and 26%, respectively (Table S3, entries 2–5). A poor conversion of 7% was obtained when using toluene as the reaction solvent, likely due to this solvent not being able to fully dissolve the ionic photosensitiser (Table S3, entry 6). Control experiments were conducted by testing the oxidation of TPP in the absence of a photosensitiser, in the dark, or under a nitrogen atmosphere to preclude oxygen from the reaction (Table S3, entries 7–11). In each case, the reaction conversion was significantly reduced, suggesting photochemical oxidation was indeed occurring. To confirm that ^1^O_2_ was the reactive oxygen species responsible for oxidising TPP to TPPO, the experiment was also conducted in the presence of DABCO, a known scavenger of ^1^O_2_ [27]. A reduced conversion of 20% was obtained, suggesting that DABCO was quenching ^1^O_2_ and preventing its reaction with TPP (Table S3, entry 12).

Oxidation of TPP was also attempted using **Ph‐DTP** as the photosensitiser—it was hypothesised that this would demonstrate negligible TPPO production due to the absence of significant light absorption around 420 nm. These reactions were carried out in toluene (5% in 15 min), DMF (4%), and acetonitrile (14%) (Table S3, entries 13–15). Unexpectedly, conversions of TPP to TPPO of 99% and 100% were obtained after 15 min of irradiation when using **Ph‐DTP** in CDCl_3_ and CHCl_3_, respectively (Table S3, entries 16 and 17). This high activity despite the almost negligible absorbance of light could be due to the significantly higher lifetime of ^1^O_2_ in these chloroform compared to the other solvents tested [28].

**FIGURE 9:**
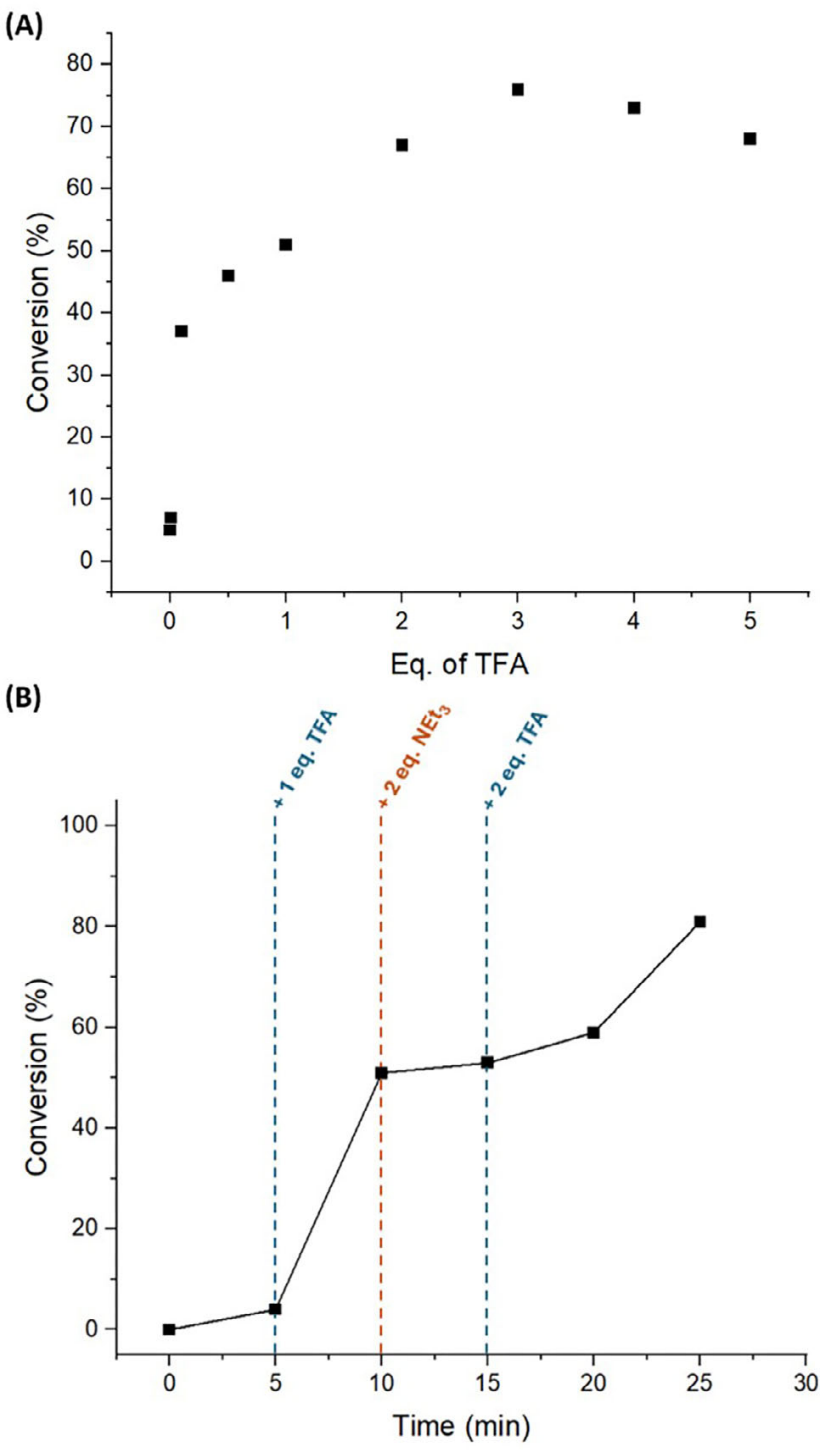
(A) Conversion of TPP to TPPO obtained using **Ph‐DTP** in the presence of different equivalents of TFA. (B) Acid–base cycling experiment showing the highest activity of **Ph‐DTP** under acidic conditions.

Photosensitisation was tested using **Ph‐DTP** in toluene in the presence of different equivalents of TFA (Table S3, entries 18–25). Toluene was selected as the solvent for these tests on the basis that it gave a low conversion and would therefore benefit from an increase in light absorption. Additionally, it did not have any Lewis basic sites (as DMF or acetonitrile did) that could complicate the initial study. In the absence of TFA, **Ph‐DTP** in toluene was able to achieve a conversion of 5% in 15 min. As the number of equivalents of TFA increased, the conversion of TPPO achieved by **Ph‐DTP** increased up to a plateau of 76% obtained after the addition of 3 equivalents (Figure 9A). This performance was significantly better than **[Ph** **‐** **DTP** **‐** **Me** ^**+**^ **][I** ^**−**^ **]** in toluene (7%, Table S3, entry 6), which could in part be explained by the complete solubility of **Ph‐DTP** and **Ph‐DTP‐H** ^**+**^ in toluene. As a control experiment, the oxidation of TPP was also tested without a photosensitiser present in toluene without TFA and with 5 equivalents of TFA added as an excess (Table S3, entries 26 and 27). Conversions of 0% and 8% were obtained after 15 min, respectively, indicating that TFA had little effect on TPP by itself. The role of the acid in the reaction was further investigated by a time‐course experiment in which the reaction was sequentially doped with TFA (1 equivalent) followed by triethylamine (2 equialents), then TFA (2 equivalents) (Figure 9B). The rate of the reaction was quickest following the addition of TFA and stalled upon the addition of triethylamine, suggesting deprotonation of **Ph‐DTP‐H^+^** turned off the photoactivity.

**FIGURE 10:**
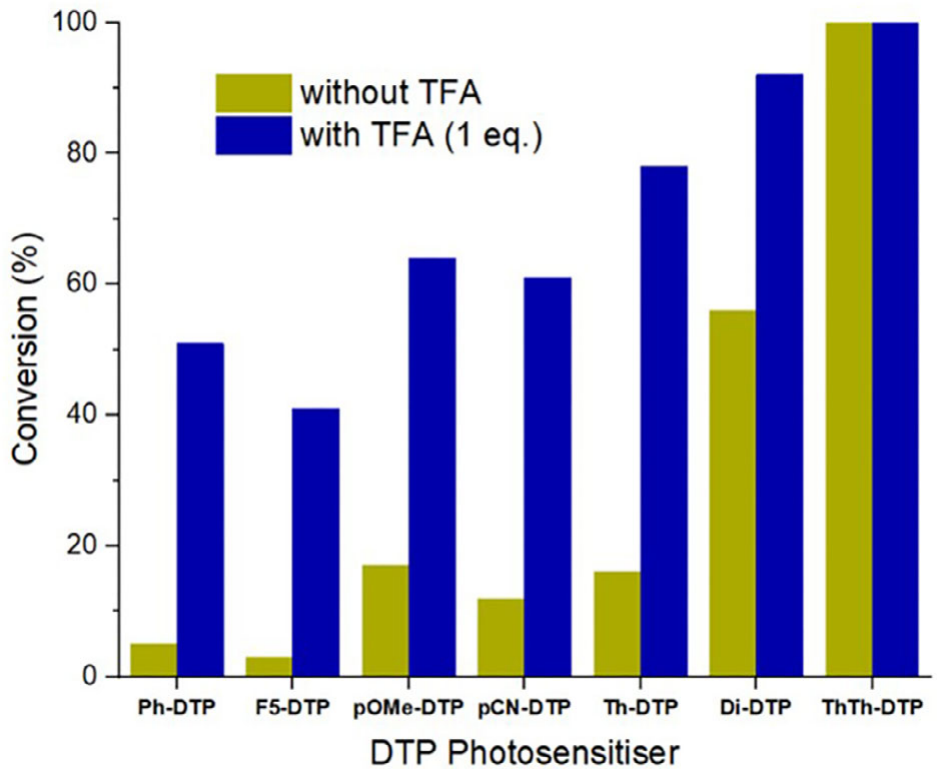
Comparison of the conversions of TPP to TPPO by each **DTP** photosensitiser in toluene solution with and without TFA under 420 nm light.

Having established that TFA could turn on the photosensitising properties of the **Ph‐DTP**, the activity of the remaining **DTP** scope was evaluated with and without acid present using toluene and acetonitrile as solvents (Table S4 and Figure 10). In all cases, the activity of the **DTP** was significantly enhanced by the addition of 1 equivalent of TFA. For **Ph‐DTP**, **F5‐DTP**, **pOMe‐DTP**, **pCN‐DTP**, and **Th‐DTP**, the experiments conducted in toluene in the absence of acid resulted in low reaction conversions (3%–17%), rising to 41%—78% after the addition of 1 equivalent of TFA (Table S4, entries 1–20). Overall, the reactions conducted in acetonitrile displayed higher initial conversions (14%–60%) but still rose significantly upon protonation (87%–100%). These results are directly comparable to the performance of **[Ph** **‐** **DTP** **‐** **Me** ^**+**^ **][I** ^**−**^ **]** in acetonitrile, which resulted in a conversion of 85% after 15 min (Table S3, entry 4). Each of these **DTPs** was fully soluble in both neutral and acidified toluene, unlike **[Ph** **‐** **DTP** **‐** **Me** ^**+**^ **]** **[I** ^**−**^ **]**, which was insoluble in toluene. For the **DTPs** doped with 1 equivalent of TFA, the conversions of TPP to TPPO exhibited a positive correlation with the amount of light each **DTP** absorbed around the 400–420 nm region. The most extreme examples were **Di‐DTP** and **ThTh‐DTP**, which achieved 92% and 100% conversion in toluene with 1 equivalent of TFA (Table S4, entries 22 and 26). The performance with and without TFA of **Ph‐DTP** was also tested in DMF, resulting in conversions of 4% and 29%, respectively (Table S3, entries 14 and 28). An explanation for the relatively minor improvement in conversion in acidic DMF is the Lewis basic sites of the solvent molecules competing with **Ph‐DTP** for the TFA: in comparison, acetonitrile is a much weaker Lewis base [29]. Under 420 nm light, **ThTh‐DTP** and **Di‐DTP** both achieved > 95% conversion in acetonitrile without any acid being added due to the significant light absorption exhibited by these two dyes at the wavelength used (Table S4, entries 23 and 28). To further test **ThTh‐DTP**, the oxidation was carried out using 500 nm light both with and without acid present in the toluene solution. The result was 21% conversion without adding TFA and 58% in the presence of 1 equivalent of TFA (Table S4, entries 29 and 30).

Other examples of acid‐triggered photosensitisers have been previously presented in literature, with different methods of operation highlighted [30]. For example, Zhang and coworkers have reported on a BODIPY‐based photosensitiser featuring a diethylamine group [31]. In this example, protonation of the diethylamine group suppressed photoinduced electron transfer that would otherwise deactivate the excited state photosensitiser before triplet–triplet annihilation could occur. Other reported mechanisms include pH‐triggered disassembly of self‐aggregated photosensitisers and supramolecular structures: in the aggregated form, the photoactivity is quenched and only activates upon disassembly in acidic environments [32, 33]. Mechanisms such as these have been utilised in pH‐regulated photodynamic therapy at acidic tumor microenvironments [34, 35]. Beyond protonation‐triggered onset of photoactivity, both Rickertsen et al. and Reich et al. have demonstrated that complexation of acridine derivatives to Lewis acids such as Yb(OTf)_3_, Sc(OTf)_3_, or BF_3_.OEt_2_ yielded potent photoredox catalysts [22, 36].

## 3 Conclusion

In conclusion, a library of **DTPs** has been synthesised using the straightforward reaction between an aromatic aldehyde and 3‐aminothiophene oxalate, and their optoelectronic properties were subsequently investigated both experimentally and computationally. While the neutral **DTPs** mainly absorbed UV light, protonation using TFA or methylation using methyl iodide induced bathochromic shifts such that the charged dyes exhibited absorption of visible light. In the case of the double **Di‐DTP**, single protonation (**Di‐DTP‐** **H** ^**+**^) led to a dipolar electron donor‐acceptor type structure and induced a bathochromic shift in emission. Protonation of the second **DTP** group (**Di‐DTP‐2** **H** ^**+**^) reduced this dipolar character, reverting the lowest energy transition to *π*–π* and producing a hypsochromic shift in emission relative to **Di‐DTP‐** **H** ^**+**^. The shift in absorption wavelengths toward visible light upon protonation allowed the photosensitising activities of the **DTPs** to be controlled under 420 nm irradiation: an up to 13.5‐fold increase in activity was observed upon the addition of 1 equivalent of TFA.

## Conflicts of Interest

The authors declare no conflicts of interest.

## Supporting information



The authors have cited additional references within the Supporting Information [37, 38, 39, 40, 41, 42, 43, 44, 45].
**Supporting File**: chem71186‐sup‐0001‐SuppMat.docx.

## Data Availability

The data that support the findings of this study are available in the Supporting Information of this article and from the Cambridge Structural Database (CCDC deposition numbers 2503850‐2503853).
