# Supplementary material for: Single‐Step Synthesis of Dithieno[3,2‐b:2′,3′‐e]Pyridines and Their Acid‐Dependent Optical and Photosensitising Properties
Source: Chemistry. 2026 May 27;32(34):e71186. doi: 10.1002/chem.71186 (PMC13569972; doi:10.1002/chem.71186)
Supplement: Supplementary file 1 [file CHEM-32-e71186-s001.docx]

**Single-Step Synthesis of Dithieno[3,2-*b*:2’,3’-*e*]pyridines and Their Acid-Dependent Optical and Photosensitising Properties**

**Electronic Supplementary Information**

Dominic Taylor,^a^ Matthew Wallace,^a,b^ Leonardo Amicosante,^b^ Gary S. Nichol,^a^ Scott J. Dalgarno,^b^ Filipe Vilela, ^b,^* Iain A. Wright, ^a,^* and Neil B. McKeown.^a,^*

^a^EaStCHEM School of Chemistry, University of Edinburgh, Edinburgh, EH9 3FJ, UK.

^b^School of Engineering and Physical Sciences, Heriot-Watt University, Riccarton, Edinburgh, EH14 4AS, UK.

*Corresponding author.

E-mail: neil.mckeown@ed.ac.uk, iain.wright@ed.ac.uk, f.vilela@hw.ac.uk

**Table of Contents**

| **1** | **General Experimental Details** | 2 |
| --- | --- | --- |
| **2** | **Synthesis and Spectroscopic Characterisation** | 4 |
| **3** | **Single Crystal X-Ray Structures** | 11 |
| **4** | **UV-Vis Absorption and Emission Data** | 15 |
| **5** | **Photosensitising Studies** | 26 |
| **6** | **DFT Calculations** | 30 |
| **7** | **Spectra of Synthesised Compounds** | 35 |
| **8** | **References** | 58 |

**1.	General Experimental Details**

All reagents and starting materials utilised in this work were commercially available and used without further purification.

^1^H, ^13^C and ^19^F NMR spectra of all compounds were recorded using a 500 MHz Bruker AVIIIHD spectrometer at ambient temperature in CDCl_3_ solution using the residual CHCl_3_ peak as the internal reference. IR spectra of synthesised compounds was recorded on powdered samples using a Shimadzu IRAffinity-1S Fourier Transform Infrared Spectrophotometer. Melting points were recorded using a Stuart SMP10 melting point apparatus using glass capillary tubes and are uncorrected. High resolution mass spectra (HRMS) were recorded using a Thermo/Finnigan MAT 900 mass spectrometer using electrospray ionisation (ESI).

Solution-state UV-Vis absorption spectra were measured in CHCl_3_ solution using a PerkinElmer Lambda 25 spectrometer with 10 mm quartz cuvettes at known concentrations. Solution-state emission spectra were recorded in CHCl_3_ solution using a Shimadzu RF-6000 Spectro Fluorophotometer using 10 mm quartz cuvettes. Molar extinction coefficients (*ε*) were measured using a series of stock solutions of **DTP** in chloroform. The coefficient was extracted as the gradient of the resulting Beer-Lambert plots with the quoted error being the error arising from the linear regression analysis. The absorbance data is presented as either *ε* or normalised where appropriate.	The absolute PLQY of **Ph-DTP** was measured following the considerations outlined by Jones *et al*. using a FLS920 spectrophotometer (from Edinburgh Instruments Ltd.), extended red-sensitive photon multiplier detector with a 750 nm detection grating (R2658P, Hamamatsu) and an integrating sphere of 102 mm inner diameter (Yobin Yvon).^1^ The sample of **Ph-DTP** was a 200 µM solution in CHCl_3_ in a square 1 cm path length quartz cuvette placed in the center of the integrating sphere: the reference solution was pure CHCl_3_. Excitation was achieved using a 450 W Xenon Lamp (Xe2, Edinburgh Instruments Ltd) with a 300 nm excitation grating: the excitation wavelength was 330 nm with a bandwidth of 5 nm. The dwell time was 0.2 s and emission was measured from 340 – 540 nm in 2 nm intervals.

Single crystal X-ray diffraction was performed by mounting suitable crystals using a MITIGEN holder in paratone oil on a Bruker D8 Venture diffractometer. The crystal was kept at a steady *T* = 100.00 K during data collection. The structure was solved with the ShelXS solution program using direct methods and by using Olex2 1.5-beta as the graphical interface.^2,3^ The model was refined with olex2.refine 1.5-beta using full matrix least squares minimisation on *F*^2^.^4^ CCDC 2503850 - 2503854 contains the supplementary crystallographic data for this paper. These data can be obtained free of charge from The Cambridge Crystallographic Data Centre *via* www.ccdc.cam.ac.uk/structures.

Photochemistry experiments were conducted using LED modules constructed in house. Each module consisted of 4 x 3 W LEDs (either 410 – 420 or 500 – 510 nm) purchased from Future Eden. The LEDs were mounted onto 100 x 100 x 40 mm aluminium heatsinks purchased from RS Components Ltd using thermal paste and powered using a 600 mA 18-34 V LED driver power supply as previously described.^5^ The conversion obtained during photochemical reactions was measured using quantitative ^31^P NMR spectroscopy on a Bruker AVIIIHD 400 MHz spectrometer in CDCl_3_: spectra were recorded using an inverse gated sequence with a D_1_ of 40 s and an acquisition time of 2.5 seconds.^6^ The reaction conversion was calculated directly from the ratio of TPPO to TPP integrals.

**2.	Synthesis and Spectroscopic Characterisation**

**Synthesis of 3-Aminothiophene Oxalate**

Adapted from literature procedure by Barker *et al*.^7^ Methyl 3-amino-2-thiophenecarboxylate (31.4 g, 200 mmol) was refluxed in a solution of sodium hydroxide (20 g, 500 mmol) in water (100 mL) for 30 minutes. The mixture was cooled to room temperature then with ice, then acidified slowly with concentrated hydrochloric acid until the mixture tested *ca*. pH 3 with universal indicator paper. The precipitate was filtered and then pressed dry between sheets of filter paper. The resultant paste was reacted in 1-propanol (80 mL) with anhydrous oxalic acid (19.8 g, 220 mmol) at 40 ^o^C for 45 minutes. After cooling, the mixture was diluted with diethyl ether, the solid filtered off and washed with additional diethyl ether to yield white crystals (32.1 g, 85%). Product was in used **DTP** synthesis without further purification.

**General Procedure for DTP Synthesis**

3-Aminothiophene oxalate (1.89 g, 10 mmol) and aromatic aldehyde (5 mmol) dissolved in DCM (30 mL) were cooled with an ice-bath then TFA (30 µL) was added slowly. The mixture was stirred at room temperature for 90 minutes then TFA (765 µL, 10 mmol) added and the reaction heated to reflux overnight. After cooling to room temperature, the reaction mixture was diluted with diethyl ether (50 mL) and water (50 mL). The aqueous layer was basified with 1 M NaOH (aq) then extracted with diethyl ether (2 x 50 mL). The combined organic layers were dried over MgSO_4_ and the solvent removed under reduced pressure. Purification achieved as stated below.

**Ph-DTP**

General procedure using benzaldehyde (531 mg, 511 µL, 5 mmol). Recrystallised from hot acetone to give bright yellow/green crystals (300 mg, 22%). **^1^H NMR** (CDCl_3_, 500 MHz, 25 ^o^C) δ_H_ 7.85 (m, 2 *H*), 7.79 (d, J = 5.7 Hz, 2 *H*), 7.65 (d, J = 5.7 Hz, 2 *H*), 7.60 (m, 2 *H*), 7.55 (m, 1 *H*). **^13^C NMR** (CDCl_3_, 125 MHz, 25 ^o^C) δ_C_ 156.2, 138.7, 137.0, 131.6, 129.7, 129.3, 129.2, 128.1, 125.1. **IR** $\bar{\nu}$ (cm^−1^) 3068 (C-H str., w.). **UV-Vis** (CHCl_3_) λ_max_ (nm) 330, 260. **MP** (^o^C) 173 – 175. **HRMS** (ESI+) m/z 268.0245 [M+H]^+^, calc. 268.0249.

**F5-DTP**

General procedure using pentafluorobenzaldehyde (981 mg, 5 mmol). Recrystallised from hot ethanol to give dull green crystals (424 mg, 24%). **^1^H NMR** (CDCl_3_, 500 MHz, 25 ^o^C) δ_H_ 7.82 (d, J = 5.6 Hz, 2 *H*), 7.70 (d, J = 5.6 Hz, 2 *H*). **^13^C NMR** (CDCl_3_, 125 MHz, 25 ^o^C) δ_C_ 155.9, 131.4, 125.2. **^19^F NMR** (CDCl_3_, 125 MHz, 25 ^o^C) δ_F_ -137.0 (m, 2 *F*), -150.3 (m, 1 *F*), -159.1 (m, 2 *F*). **IR** $\bar{\nu}$ (cm^−1^) 3059 (C-H str., w.). **UV-Vis** (CHCl_3_) λ_max_ (nm) 332, 258. **MP** (^o^C) 154 – 156. **HRMS** (ESI+) m/z 357.9786 [M+H]^+^, calc. 357.9778.

**pCN-DTP**

General procedure using 4-cyanobenzaldehyde (656 mg, 5 mmol). Washed with hot acetone to give a beige powder (545 mg, 37%). **^1^H NMR** (CDCl_3_, 500 MHz, 25 ^o^C) δ_H_ 7.99 (m, 2 *H*), 7.91 (m, 2 *H*), 7.83 (d, J = 5.6 Hz, 2 *H*), 7.68 (d, J = 5.6 Hz, 2 *H*). **^13^C NMR** (CDCl_3_, 125 MHz, 25 ^o^C) δ_C_ 156.4, 141.4, 136.4, 133.2, 131.7, 129.0, 128.8, 125.3, 118.2, 113.6. **IR** $\bar{\nu}$ (cm^−1^). 3090 (C-H str., w.), 2228 (C≡N str., m.). **UV-Vis** (CHCl_3_) λ_max_ (nm) 335. **MP** (^o^C) 251 – 252. **HRMS** (ESI+) m/z 293.0203 [M+H]^+^, calc. 293.0202.

**pOMe-DTP**

General procedure using 4-methoxybenzaldehyde (681 mg, 5 mmol). Recrystallised from hot acetone to give bright yellow crystals (320 mg, 22%). **^1^H NMR** (CDCl_3_, 500 MHz, 25 ^o^C) δ_H_ 7.83 (m, 2 *H*), 7.80 (d, J = 5.6 Hz, 2 *H*), 7.64 (d, J = 5.6 Hz, 2 *H*), 7.13 (m, 2 *H*), 3.93 (s, 3 *H*). **^13^C NMR** (CDCl_3_, 125 MHz, 25 ^o^C) δ_C_ 160.6, 156.2, 138.6, 131.5, 129.5, 129.3, 129.2, 125.2, 114.7, 55.4. **IR** $\bar{\nu}$ (cm−1 ) 3078 (C-H str., w.), 3008 (C-H str., w.). **UV-Vis** (CHCl_3_) λ_max_ (nm) 331, 263. **MP** (^o^C) 171 – 172. **HRMS** (ESI+) m/z 298.0354 [M+H]^+^, calc. 298.0355.

**Th-DTP**

General procedure using 2-thiophenecarboxaldehyde (561 mg, 468 µL, 5 mmol). Recrystallised from hot acetone to give bright yellow crystals (277 mg, 21%). **^1^H NMR** (CDCl_3_, 500 MHz, 25 ^o^C) δ_H_ 7.90 (dd, J = 3.7, 1.1 Hz, 1 *H*), 7.83 (d, J = 5.7 Hz, 2 *H*), 7.65 (d, J = 5.7 Hz, 2 *H*), 7.64 (dd, J = 5.1, 1.1 Hz, 1 *H*), 7.32 (dd, J = 5.1, 3.7 Hz, 1 *H*). **^13^C NMR** (CDCl_3_, 125 MHz, 25 ^o^C) δ_C_ 156.3, 138.0, 132.0, 131.5, 129.2, 128.4, 128.0, 128.0, 125.3. **IR** $\bar{\nu}$ (cm^−1^) 3045 (C-H str., w.). **UV-Vis** (CHCl_3_) λ_max_ (nm) 342, 265. **MP** (^o^C) 148 - 150. **HRMS** (ESI+) m/z 273.9815 [M+H]^+^, calc. 273.9813.

**Di-DTP**

General procedure using terephthaldehyde (671 mg, 5 mmol), 3-aminothiophene oxalate (3.784 g, 20 mmol) and trifluoroacetic acid (1.5 mL, 20 mmol). Instead of extraction with diethyl ether, the crude product was stirred in hot ethanol then hot acetone. Filtration yielded an orange powder (1.488 g, 65%). **^1^H NMR** (CDCl_3_, 500 MHz, 25 ^o^C) δ_H_ 8.15 (s, 4 *H*), 7.87 (d, J = 5.7 Hz, 4 *H*), 7.71 (d, J = 5.7 Hz, 4 *H*). **^13^C NMR** (CDCl_3_, 125 MHz, 25 ^o^C) δ_C_ 131.7, 129.1, 125.3. **IR** $\bar{\nu}$ (cm^−1^) 3059 (C-H str., w.). **UV-Vis** (CHCl_3_) λ_max_ (nm) 331, 259. **IR** $\bar{\nu}$ (cm^−1^) 2987 (C-H str., w.). **MP** (^o^C) > 300. **HRMS** (ESI+) m/z 456.9945 [M+H]^+^, calc. 456.9956.

**ThBr-DTP**

General procedure using 5-bromo-2-thiophenecarboxaldehyde (955 mg, 594 µL, 5 mmol). Washed in hot ethanol to give a dull yellow powder (240 mg, 14%). **^1^H NMR** (CDCl_3_, 500 MHz, 25 ^o^C) δ_H_ 7.83 (d, J = 5.7 Hz, 2 *H*), 7.65 (d, J = 5.7 Hz, 2 *H*), 7.63 (d, J = 3.9 Hz, 2 *H*), 7.27 (d, J = 3.9 Hz, 2 *H*). **^13^C NMR** (CDCl_3_, 125 MHz, 25 ^o^C) δ_C_ 156.3, 139.4, 131.5, 130.9, 130.8, 129.5, 128.1, 125.4, 115.7. **IR** $\bar{\nu}$ (cm^−1^) 3082 (C-H str., w.). **MP** (^o^C) 193 - 196. **HRMS** (ESI+) m/z 351.8913 [M+H]^+^, calc. 351.8919.

**ThTh-DTP**

**ThBr-DTP** (0.0936 g, 0.320 mmol), 2-thiophene boronic acid pinacol ester (0.0894 g, 0.647 mmol) and K_2_CO_3_ (0.1684 g, 0.802 mmol) were mixed in a two necked round bottomed flask and cycled (3 times) with a Schlenk line under N_2_ atmosphere. Pd(PPh_3_)_4_ (0. 017 g, 0.015 mmol) was added to the two necked flask and cycled (3 times). Degassed tetrahydrofuran (80 mL) and water (10 mL) were added then the reaction was heated under N_2_ for 2 days at 70 ^o^C. After cooling to room temperature the product was extracted using DCM and washed with water. The organic layer collected and dried over MgSO_4_, then the solvent removed under reduced pressure. The product was purified by stirring the product in hot hexane then filtering to get the product as a yellow powder (70 mg, 62%). **^1^H NMR** (CDCl_3_, 500 MHz, 25 ^o^C) δ_H_ 7.84 (d, J = 5.5 Hz, 2 *H*), 7.83 (d, J = 3.5 Hz, 1 *H*), 7.66 (d, J = 5.5 Hz, 2 *H*), 7.36 (d, J = 4.0 Hz, 1 *H*), 7.33 (dd, J = 3.5, 1.0 Hz, 1 *H*), 7.32 (dd, J = 5.0, 1.0 Hz, 1 *H*), 7.09 (dd, J = 5.0, 3.5 Hz, 1 *H*). **^13^C NMR** (CDCl_3_, 125 MHz, 25 ^o^C) δ_C_ 156.3, 140.2, 136.4, 136.4, 131.6, 131.4, 130.1, 128.1, 128.0, 125.6, 125.4, 124.8, 124.4. **IR** $\bar{\nu}$ (cm^−1^) 3053 (C-H str., m.). **UV-Vis** (CHCl_3_) λ_max_ (nm) 378, 266. **MP** (^o^C) 167-169. **HRMS** (ESI+) m/z 355.9699 [M+H]^+^, calc. 355.9691.

**[Ph-DTP-Me^+^][I^-^]**

**Ph-DTP** (534 mg, 2 mmol) was dissolved in DCM (25 mL) then methyl iodide (4.96 mL, 60 mmol) added. The mixture was stirred at room temperature for 8 days then the mixture filtered and washed with a small volume of DCM. The resulting solid was dried under air to yield a bright yellow powder (641 mg, 79%). **^1^H NMR** (CDCl_3_, 500 MHz, 25 ^o^C) δ_H_ 8.54 (d, J = 5.8 Hz, 2 *H*), 8.34 (d, J = 5.8 Hz, 2 *H*), 7.88 (m, 2 *H*), 7.73 (m, 2 *H*), 7.73 (m, 1 *H*), 5.09 (s, 3 *H*). **^13^C NMR** (CDCl_3_, 125 MHz, 25 ^o^C) δ_C_ 147.3, 142.6, 133.6, 132.6, 132.3, 130.1, 128.3, 119.1, 44.9. **IR** $\bar{\nu}$ (cm^−1^) 3038 (C-H str., w.). **UV-Vis** (CHCl_3_) λ_max_ 373, 340, 298 (nm). **MP** (^o^C) 273 – 274. **HRMS** (ESI+) m/z 282.0414 [M]^+^, calc. 282.0406. **HRMS** (ESI-) m/z 126.9046 [M]^-^, calc. 126.9050.

**[Ph-ACR-Me^+^][I^-^]**

**Ph-ACR** (534 mg, 2 mmol) was dissolved in DCM (25 mL) then methyl iodide (4.96 mL, 60 mmol) added. The mixture was stirred at room temperature for 28 days then the solvent and excess methyl iodide removed under reduced pressure to yield a purple solid (675 mg, 85%). **^1^H NMR** (CDCl_3_, 500 MHz, 25 ^o^C) δ_H_ 8.94 (d, J = 9.3 Hz, 2 *H*), 8.42 (ddd, J = 9.3, 3.4, 1.5 Hz, 2 *H*), 8.01 (dd, J = 8.7, 1.2 Hz, 2 *H*), 7.81 (ddd, J = 8.7, 6.8, 0.8 Hz, 2 *H*), 7.74 (m, 2 *H*), 7.72 (m, 1 *H*), 7.50 (m, 2 *H*), 5.27 (s, 3 *H*). **^13^C NMR** (CDCl_3_, 125 MHz, 25 ^o^C) δ_C_ 161.3, 141.6, 139.2, 133.0, 130.5, 130.2, 129.8, 129.1, 128.0, 126.2, 119.6, 41.5. **IR** $\bar{\nu}$ (cm^−1^) 3034 (C-H str., w.), 2974 (C-H str., w.). **UV-Vis** (CHCl_3_) λ_max_ 425, 363, 348 (nm). **MP** (^o^C) 225 - 227. **HRMS** (ESI+) m/z 270.1271 [M]^+^, calc. 270.1277. **HRMS** (ESI-) m/z 126.9048 [M]^-^, calc. 126.9050.

**Figure S1** Comparison of the partial ^1^H NMR spectra of **Ph-DTP** (bottom) and **Ph-DTP** doped with 1 equivalent of trifluoroacetic acid (top) in CDCl_3_ showing the shift in the thiophene *α*,*β*-proton peaks upon *N*-protonation.

**Figure S2** Comparison of the partial ^1^H NMR spectra of **Ph-DTP** (bottom) and **[Ph-DTP-Me^+^][I^-^]** (top) in CDCl_3_ showing the shift in the thiophene *α*,*β*-proton peaks upon *N*-methylation.

**Figure S3** Comparison of the partial ^1^H NMR spectra of **Ph-ACR** (bottom) and **[Ph-ACR-Me^+^][I^-^]** (top) in CDCl_3_ showing the shift in the thiophene *α*,*β*-proton peaks upon *N*-methylation.

**3.	Single Crystal X-Ray Structures**

**Crystal Data for Ph-DTP (CCDC Deposition Number - 2503850)**

Grown by slow evaporation from THF solution.

**Figure S4** Single crystal X-ray structure of **Ph-DTP**. The atoms are shown as thermal ellipsoids at 50% probability. Hydrogen atoms removed for clarity.

**Crystal Data for F5-DTP (CCDC Deposition Number - 2503851)**

Grown by slow evaporation from THF solution.

**Figure S5** Single crystal X-ray structure of **F5-DTP**. The atoms are shown as thermal ellipsoids at 50% probability. Hydrogen atoms removed for clarity.

**Crystal Data for pOMe-DTP (CCDC Deposition Number - 2503852)**

Grown by slow evaporation from acetone solution.

**Figure S6** Single crystal X-ray structure of **pOMe-DTP**. The atoms are shown as thermal ellipsoids at 50% probability. Hydrogen atoms removed for clarity.

**Crystal Data for Th-DTP (CCDC Deposition Number - 2503853)**

Grown by slow evaporation from CHCl_3_ solution.

**Figure S7** Single crystal X-ray structure of **Th-DTP**. The atoms are shown as thermal ellipsoids at 50% probability. Hydrogen atoms removed for clarity.

**Crystal Data for [Ph-DTP-Me^+^][I^-^] (CCDC Deposition Number - 2503854)**

Grown by slowly cooling a saturated solution of [Ph-DTP-Me^+^][I^-^] in hot acetonitrile.

**Figure S8** Single crystal X-ray structure of **[Ph-DTP-Me^+^][I^-^]**. The atoms are shown as thermal ellipsoids at 50% probability. Hydrogen atoms removed for clarity.

Table S1 Crystal data for all single crystal X-ray structures.

| Compound | Ph-DTP | F5-DTP | pOMe-DTP | Th-DTP | [Ph-DTP-Me^+^][I^-^] |
| --- | --- | --- | --- | --- | --- |
| Formula | C_15_H_9_NS_2_ | C_15_H_4_NF_5_S_2_ | C_16_H_11_NOS_2_ | C_13_H_7_NS_3_ | C_16_H_12_INS_2_ |
| *D_calc._*/ g cm^-3^ | 1.474 | 1.763 | 1.462 | 1.607 | 1.825 |
| *m*/mm^-1^ | 0.419 | 0.450 | 3.514 | 0.627 | 19.401 |
| Formula Weight | 267.377 | 357.330 | 297.404 | 273.405 | 409.317 |
| Colour | colourless | colourless | clear colourless | yellow | translucent dark yellow |
| Shape | block-shaped | block-shaped | plate-shaped | block-shaped | prism-shaped |
| Size/mm^3^ | 0.29×0.26×0.20 | 0.33×0.14×0.14 | 0.19×0.16×0.05 | 0.41×0.20×0.18 | 0.41×0.13×0.07 |
| *T*/K | 100.00(10) | 100.00 | 100.00(10) | 100.00 | 100.00(10) |
| Crystal System | monoclinic | monoclinic | monoclinic | monoclinic | monoclinic |
| Space Group | *C*2/*c* | *P*2_1_/*c* | *P*2_1_/*c* | *P*2_1_/*n* | *P*2_1_/*n* |
| *a*/Å | 15.6821(3) | 10.6651(3) | 11.95067(14) | 10.0536(3) | 6.70653(3) |
| *b*/Å | 7.16251(13) | 17.5039(6) | 18.1434(2) | 7.1930(2) | 13.50664(6) |
| *c*/Å | 22.1969(3) | 15.1317(5) | 6.33372(7) | 15.6256(5) | 16.44556(7) |
| *α*/^°^ | 90 | 90 | 90 | 90 | 90 |
| *β*/^°^ | 104.8702(14) | 107.5984(11) | 100.4105(11) | 91.1073(10) | 90.8718(4) |
| *γ*/^°^ | 90 | 90 | 90 | 90 | 90 |
| V/Å^3^ | 2409.73(7) | 2692.60(15) | 1350.71(3) | 1129.76(6) | 1489.511(11) |
| *Z* | 8 | 8 | 4 | 4 | 4 |
| *Z'* | 1 | 2 | 1 | 1 | 1 |
| Wavelength/Å | 0.71073 | 0.71073 | 1.54184 | 0.71073 | 1.54184 |
| Radiation type | Mo K*_a_* | Mo K*_a_* | Cu K*_a_* | Mo K*_a_* | Cu K*_a_* |
| *Θ_min_*/^°^ | 2.87 | 2.32 | 3.76 | 2.39 | 4.24 |
| *Θ_max_*/^°^ | 45.50 | 33.18 | 78.27 | 37.30 | 77.12 |
| Measured Refl's. | 53208 | 115203 | 33728 | 54537 | 61292 |
| Indep't Refl's | 10118 | 10273 | 2865 | 5583 | 3147 |
| Refl's I≥2*σ*(I) | 8198 | 8828 | 2861 | 4946 | 3147 |
| *R*_int_ | 0.0291 | 0.0425 | 0.0262 | 0.0356 | 0.0458 |
| Parameters | 244 | 487 | 280 | 217 | 290 |
| Restraints | 0 | 0 | 0 | 0 | 270 |
| Largest Peak | 0.4131 | 0.2958 | 0.1087 | 0.2962 | 0.8367 |
| Deepest Hole | -0.3804 | -0.2470 | -0.2096 | -0.3307 | -0.8932 |
| GooF | 1.0084 | 1.0689 | 1.0732 | 1.1041 | 1.0111 |
| *wR_2_* (all data) | 0.0420 | 0.0295 | 0.0354 | 0.0266 | 0.0416 |
| *wR_2_* | 0.0395 | 0.0268 | 0.0354 | 0.0251 | 0.0416 |
| *R_1_* (all data) | 0.0318 | 0.0257 | 0.0132 | 0.0243 | 0.0164 |
| *R_1_* | 0.0219 | 0.0177 | 0.0132 | 0.0175 | 0.0164 |

4.	UV-Vis Absorption and Emission Data

**Figure S9** UV-Vis absorption (black solid line) and normalised emission (blue dashed line) spectra of **Ph-DTP** in CHCl_3_ solution at a concentration of 100 µM.

**Figure S10** UV-Vis absorption (black solid line) and normalised emission (blue dashed line) spectra of **F5-DTP** in CHCl_3_ solution at a concentration of 100 µM.

**Figure S11** UV-Vis absorption (black solid line) and normalised emission (blue dashed line) spectra of **pCN-DTP** in CHCl_3_ solution at a concentration of 100 µM.

**Figure S12** UV-Vis absorption (black solid line) and normalised emission (blue dashed line) spectra of **pOMe-DTP** in CHCl_3_ solution at a concentration of 100 µM.

**Figure S13** UV-Vis absorption (black solid line) and normalised emission (blue dashed line) spectra of **Th-DTP** in CHCl_3_ solution at a concentration of 100 µM.

**Figure S14** UV-Vis absorption (black solid line) and normalised emission (blue dashed line) spectra of **Di-DTP** in CHCl_3_ solution at a concentration of 100 µM.

**Figure S15** UV-Vis absorption (black solid line) and normalised emission (blue dashed line) spectra of **ThTh-DTP** in CHCl_3_ solution at a concentration of 100 µM.

**Figure S16** Normalised UV-Vis absorption (solid line) and normalised emission (dashed line) spectra of **Ph-DTP** in various solvents at a concentration of 100 µM.

**Figure S17** Lippert-Mataga plot of Stokes’ shift against orientation polarizability function (ΔF). R^2^ = 0.86.

**Table S2** Data used to construct the Lippert-Mataga plot for **Ph-DTP** shown in Figure S14. ^a^Calculated from equation (1). ^b^Values for n and *ε* from reference S8.

| **Solvent** | **λ_abs_/nm** | **λ_em_/nm** | **Stokes Shift (Δ*ν*)/ x 10^3^ cm^-1^** | **Δf^a,b^** |
| --- | --- | --- | --- | --- |
| Hexane | 328 | 359 | 2.63 | 0.0012 |
| Ethyl Acetate | 328 | 368 | 3.31 | 0.148 |
| DCM | 329 | 372 | 3.51 | 0.200 |
| THF | 328 | 369 | 3.39 | 0.210 |
| Methanol | 328 | 377 | 3.96 | 0.218 |
| Acetonitrile | 327 | 372 | 3.70 | 0.263 |
| DMSO | 330 | 380 | 3.99 | 0.305 |
| Chloroform | 330 | 372 | 3.42 | 0.309 |

The orientation polarizability function, Δf, can be defined as in equation (1):

$\Delta f(\varepsilon, n)=\left( \frac{\varepsilon-1}{2\varepsilon+1} \right)-\left( \frac{n^{2}-1}{2n^{2}+1} \right)$	(1)

where *ε* is the dielectric constant and n is the refractive index of each solvent.

**Figure S18** UV-Vis absorption spectra of **Ph-DTP** and **[Ph-DTP-Me^+^][I^-^]** in CHCl_3_ solution at a concentration of 100 µM.

**Figure S19** UV-Vis absorption spectra (left) and emission (right) spectra of **Ph-DTP** in CHCl_3_ solution at a concentration of 100 µM in the presence of 0 – 100 equivalents of trifluoroacetic acid.

**Figure S20** UV-Vis absorption spectra (left) and emission (right) spectra of **F5-DTP** in CHCl_3_ solution at a concentration of 100 µM in the presence of 0 – 100 equivalents of trifluoroacetic acid.

**Figure S21** UV-Vis absorption spectra (left) and emission (right) spectra of **pOMe-DTP** in CHCl_3_ solution at a concentration of 100 µM in the presence of 0 – 100 equivalents of trifluoroacetic acid.

**Figure S22** UV-Vis absorption spectra (left) and emission (right) spectra of **pCN-DTP** in CHCl_3_ solution at a concentration of 100 µM in the presence of 0 – 100 equivalents of trifluoroacetic acid.

**Figure S23** UV-Vis absorption spectra (left) and emission (right) spectra of **Th-DTP** in CHCl_3_ solution at a concentration of 100 µM in the presence of 0 – 100 equivalents of trifluoroacetic acid.

**Figure S24** UV-Vis absorption spectra (left) and emission (right) spectra of **Di-DTP** in CHCl_3_ solution at a concentration of 100 µM in the presence of 0 – 100 equivalents of trifluoroacetic acid.

**Figure S25** UV-Vis absorption spectra (left) and emission (right) spectra of **ThTh-DTP** in CHCl_3_ solution at a concentration of 100 µM in the presence of 0 – 100 equivalents of trifluoroacetic acid.

**Figure S26** UV-Vis absorption (black solid line) and normalised emission (blue dashed line) spectra of **Ph-ACR** in CHCl_3_ solution at a concentration of 100 µM.

**Figure S27** UV-Vis absorption spectra of **Ph-ACR** and **[Ph-ACR-Me^+^][I^-^]** in CHCl_3_ solution at a concentration of 100 µM.

**Figure S28** UV-Vis absorption spectra (left) and emission (right) spectra of 9-phenylacridine in CHCl_3_ solution at a concentration of 100 µM in the presence of 0 – 100 equivalents of trifluoroacetic acid.

5.	Photosensitising Studies

Photosensitisation Procedure

Triphenylphosphine (52.5 mg, 0.2 mmol) and DTP photosensitiser (0.01 mmol) were dissolved in solvent (5 mL) then placed 7 cm in front of a 420 nm LED. TFA (0 – 5 equivalents) was then added and the reaction was illuminated with stirring for 15 minutes. A sample of the reaction mixture was withdrawn and diluted with CDCl_3_ for analysis by ^31^P NMR spectroscopy.

**Scheme S1** Photooxidation of triphenylphosphine (TPP) to triphenylphosphine oxide (TPPO) using **DTPs**.

**Figure S29** Experimental set up for the photosensitisation of oxygen using **DTP** photosensitisers.

**Figure S30** ^31^P NMR spectra of triphenylphosphine oxide (TPPO, top), triphenylphosphine (TPP, bottom) and an example photosensitisation reaction mixture (middle) (Table S3, Entry 21, conversion – 51%).

**Table S3** Screening photosensitisation of TPP using **DTP** photosensitisers.

| **Table Entry** | **Photosensitiser** | **Solvent** | **Additive** | **Time/min** | **Conversion/%** |
| --- | --- | --- | --- | --- | --- |
| 1 | **[Ph-DTP-Me^+^][I^-^]** | CDCl_3_ | - | 15 | 87 |
| 2 | **[Ph-DTP-Me^+^][I^-^]** | CHCl_3_ | - | 15 | 85 |
| 3 | **[Ph-DTP-Me^+^][I^-^]** | DCM | - | 15 | 44 |
| 4 | **[Ph-DTP-Me^+^][I^-^]** | Acetonitrile | - | 15 | 85 |
| 5 | **[Ph-DTP-Me^+^][I^-^]** | DMF | - | 15 | 26 |
| 6 | **[Ph-DTP-Me^+^][I^-^]** | Toluene | - | 15 | 7 |
| 7 | **-** | CDCl_3_ | - | 15 | 8 |
| 8 | **[Ph-DTP-Me^+^][I^-^]** | CDCl_3_ | No light | 15 | 0 |
| 9 | **Ph-DTP** | CDCl_3_ | No light | 15 | 0 |
| 10 | **[Ph-DTP-Me^+^][I^-^]** | CDCl_3_ | No air | 15 | 5 |
| 11 | **Ph-DTP** | CDCl_3_ | No air | 15 | 8 |
| 12 | **[Ph-DTP-Me^+^][I^-^]** | Acetonitrile | DABCO (5 eq.) | 15 | 20 |
| 13 | **Ph-DTP** | Toluene | - | 15 | 5 |
| 14 | **Ph-DTP** | DMF | - | 15 | 4 |
| 15 | **Ph-DTP** | Acetonitrile | - | 15 | 14 |
| 16 | **Ph-DTP** | CHCl_3_ | - | 15 | 99 |
| 17 | **Ph-DTP** | CDCl_3_ | - | 15 | 100 |
| 18 | **Ph-DTP** | Toluene | TFA (0.01 eq.) | 15 | 7 |
| 19 | **Ph-DTP** | Toluene | TFA (0.1 eq.) | 15 | 37 |
| 20 | **Ph-DTP** | Toluene | TFA (0.5 eq.) | 15 | 46 |
| 21 | **Ph-DTP** | Toluene | TFA (1 eq.) | 15 | 51 |
| 22 | **Ph-DTP** | Toluene | TFA (2 eq.) | 15 | 67 |
| 23 | **Ph-DTP** | Toluene | TFA (3 eq.) | 15 | 76 |
| 24 | **Ph-DTP** | Toluene | TFA (4 eq.) | 15 | 73 |
| 25 | **Ph-DTP** | Toluene | TFA (5 eq.) | 15 | 68 |
| 26 | **-** | Toluene | - | 15 | 0 |
| 27 | **-** | Toluene | TFA (5 eq.) | 15 | 8 |
| 28 | **Ph-DTP** | DMF | TFA (1 eq.) | 15 | 29 |
| 29 | **Ph-DTP** | Acetonitrile | TFA (1 eq.) | 15 | 99 |

^a^Conditions: TPP (0.2 mmol), **DTP** (0.01 mmol), TFA (0 – 1 mmol), solvent (5 mL), h*ν* (420 nm LED). Reaction conversion determined by quantitative ^31^P NMR spectroscopy.

Time Course Analysis Procedure

Triphenylphosphine (52.5 mg, 0.2 mmol) and **Ph-DTP** (0.01 mmol) were dissolved in toluene (5 mL) then placed 7 cm in front of a 420 nm LED for 30 minutes. At 5 minute intervals, a sample was withdrawn and diluted with CDCl_3_ for analysis by ^31^P NMR spectroscopy. TFA was added after 5 minutes (0.1 mL of 100 mM TFA solution, 0.01 mmol) and 15 minutes (0.2 mL of 100 mM TFA solution, 0.02 mmol): triethylamine was added at the 10 minute mark (0.2 mL of 100 mM NEt_3_ solution, 0.02 mmol).

**Table S4** Testing the **DTP** photosensitiser scope using TPP oxidation.^a^

| **Table Entry** | **Photosensitiser** | **Solvent** | **Additive** | **Time/min** | **Conversion/%** |
| --- | --- | --- | --- | --- | --- |
| 1 | **Ph-DTP** | Toluene | - | 15 | 5 |
| 2 | **Ph-DTP** | Toluene | TFA (1 eq.) | 15 | 51 |
| 3 | **Ph-DTP** | Acetonitrile | - | 15 | 14 |
| 4 | **Ph-DTP** | Acetonitrile | TFA (1 eq.) | 15 | 99 |
| 5 | **F5-DTP** | Toluene | - | 15 | 3 |
| 6 | **F5-DTP** | Toluene | TFA (1 eq.) | 15 | 41 |
| 7 | **F5-DTP** | Acetonitrile | - | 15 | 59 |
| 8 | **F5-DTP** | Acetonitrile | TFA (1 eq.) | 15 | 87 |
| 9 | **pOMe-DTP** | Toluene | - | 15 | 17 |
| 10 | **pOMe-DTP** | Toluene | TFA (1 eq.) | 15 | 64 |
| 11 | **pOMe-DTP** | Acetonitrile | - | 15 | 60 |
| 12 | **pOMe-DTP** | Acetonitrile | TFA (1 eq.) | 15 | 100 |
| 13 | **pCN-DTP** | Toluene | - | 15 | 12 |
| 14 | **pCN-DTP** | Toluene | TFA (1 eq.) | 15 | 61 |
| 15 | **pCN-DTP** | Acetonitrile | - | 15 | 25 |
| 16 | **pCN-DTP** | Acetonitrile | TFA (1 eq.) | 15 | 100 |
| 17 | **Th-DTP** | Toluene | - | 15 | 16 |
| 18 | **Th-DTP** | Toluene | TFA (1 eq.) | 15 | 78 |
| 19 | **Th-DTP** | Acetonitrile | - | 15 | 52 |
| 20 | **Th-DTP** | Acetonitrile | TFA (1 eq.) | 15 | 97 |
| 21 | **Di-DTP** | Toluene | - | 15 | 56 |
| 22 | **Di-DTP** | Toluene | TFA (1 eq.) | 15 | 92 |
| 23 | **Di-DTP** | Acetonitrile | - | 15 | 95 |
| 24 | **Di-DTP** | Acetonitrile | TFA (1 eq.) | 15 | 100 |
| 25 | **ThTh-DTP** | Toluene | - | 15 | 100 |
| 26 | **ThTh-DTP** | Toluene | TFA (1 eq.) | 15 | 100 |
| 27 | **ThTh-DTP** | Acetonitrile | - | 15 | 100 |
| 28 | **ThTh-DTP** | Acetonitrile | TFA (1 eq.) | 15 | 100 |
| 29^b^ | **ThTh-DTP** | Toluene | - | 15 | 21 |
| 30^b^ | **ThTh-DTP** | Toluene | TFA (1 eq.) | 15 | 58 |

^a^Conditions: TPP (0.2 mmol), **DTP** (0.01 mmol), TFA (0 – 0.2 mmol), solvent (5 mL), h*ν* (420 nm LED). Reaction conversion determined by quantitative ^31^P NMR spectroscopy. ^b^The light source was a 500 nm LED.

6.	DFT Calculations

Computational modelling of the **DTP** compounds was carried out using density functional theory (DFT) using the Gaussian16 software package.^9^ The calculations and results were visualised using GaussView and Avogadro. Calculations were performed at the B3LYP/6-31G++(d,2p) level in a vacuum, with energetic minima confirmed by the absence of imaginary vibrational frequencies. Despite repeated attempts to optimise, **F5-DTP** displayed a low energy imaginary frequency (*ca.* -4 cm^-1^) associated with rotation of the *meso*-pentafluorophenyl group. Such low energy imaginary frequencies have been reported in literature to be a numerical artefact of the integration grid used.^10^

**Table S5** (Left) HOMO and (right) LUMO calculated for **DTP** series using DFT at the B3LYP/6-31G++(d,2p) level.

| **Molecule** | **HOMO (eV)** | **LUMO (eV)** |
| --- | --- | --- |
| **Ph-DTP** |  |  |
|  | -6.05 | -1.74 |
| **F5-DTP** |  |  |
|  | -6.46 | -2.04 |

**Table S5 (continued)**

| **Molecule** | **HOMO (eV)** | **LUMO (eV)** |
| --- | --- | --- |
| **pOMe-DTP** |  |  |
|  | -5.92 | -2.34 |
| **pCN-DTP** |  |  |
|  | -6.35 | -1.64 |
| **Th-DTP** |  |  |
|  | -6.03 | -1.93 |

**Table S5 (continued)**

| **Molecule** | **HOMO (eV)** | **LUMO (eV)** |
| --- | --- | --- |
| **ThTh-DTP** |  |  |
|  | -5.82 | -2.15 |
| **Di-DTP** |  |  |
|  | -6.09 | -2.02 |

**Table S6** (Left) HOMO and (right) LUMO calculated for protonated or *N*-methylated **DTPs** using DFT at the B3LYP/6-31G++(d,2p) level.

| **Molecule** | **HOMO (eV)** | **LUMO (eV)** |
| --- | --- | --- |
| **Ph-DTP-H^+^** |  |  |
|  | -10.11 | -6.31 |
| **[Ph-DTP-Me^+^][I^-^]** |  |  |
|  | -9.98 | -6.17 |
| **Di-DTP-H^+^** |  |  |
|  | -8.01 | -6.28 |
| **Di-DTP-2H^+^** |  |  |
|  | -12.02 | -8.30 |

**Table S6** **(continued)**

| **Molecule** | **HOMO (eV)** | **LUMO (eV)** |
| --- | --- | --- |
| **ThTh-DTP-H^+^** |  |  |
|  | -8.67 | -6.16 |

**7.	Spectra of Synthesised Compounds**

**Ph-DTP**

**Figure S31** ^1^H NMR spectra of **Ph-DTP** in CDCl_3_.

**Figure S32** ^13^C NMR spectra of **Ph-DTP** in CDCl_3_.

**Figure S33** IR spectra of **Ph-DTP**.

**Figure S34** High resolution mass spectra of **Ph-DTP**.

**F5-DTP**

**Figure S35** ^1^H NMR spectra of **F5-DTP** in CDCl_3_.

**Figure S36** ^13^C NMR spectra of **F5-DTP** in CDCl_3_.

**Figure S37** ^19^F NMR spectra of **F5-DTP** in CDCl_3_.

**Figure S38** IR spectra of **F5-DTP**.

**Figure S39** High resolution mass spectra of **F5-DTP**.

**pCN-DTP**

**Figure S40** ^1^H NMR spectra of **pCN-DTP** in CDCl_3_.

**Figure S41** ^13^C NMR spectra of **pCN-DTP** in CDCl_3_.

**Figure S42** IR spectra of **pCN-DTP**.

**Figure S43** High resolution mass spectra of **pCN-DTP**.

**pOMe-DTP**

**Figure S44** ^1^H NMR spectra of **pOMe-DTP** in CDCl_3_.

**Figure S45** ^13^C NMR spectra of **pOMe-DTP** in CDCl_3_.

**Figure S46** IR spectra of **pOMe-DTP**.

**Figure S47** High resolution mass spectra of **pOMe-DTP**.

**Th-DTP**

**Figure S48** ^1^H NMR spectra of **Th-DTP** in CDCl_3_.

**Figure S49** ^13^C NMR spectra of **Th-DTP** in CDCl_3_.

**Figure S50** IR spectra of **Th-DTP**.

**Figure S51** High resolution mass spectra of **Th-DTP**.

**ThBr-DTP**

**Figure S52** ^1^H NMR spectra of **ThBr-DTP** in CDCl_3_.

**Figure S53** ^13^C NMR spectra of **ThBr-DTP** in CDCl_3_.

**Figure S54** IR spectra of **ThBr-DTP**.

**Figure S55** High resolution mass spectra of **ThBr-DTP**.

**ThTh-DTP**

**Figure S56** ^1^H NMR spectra of **ThTh-DTP** in CDCl_3_.

**Figure S57** ^13^C NMR spectra of **ThTh-DTP** in CDCl_3_.

**Figure S58** IR spectra of **ThTh-DTP**.

**Figure S59** High resolution mass spectra of **ThTh-DTP**.

**Di-DTP**

**Figure S60** ^1^H NMR spectra of **Di-DTP** in CDCl_3_.

**Figure S61** ^13^C NMR spectra of **Di-DTP** in CDCl_3_.

**Figure S62** IR spectra of **Di-DTP**.

**Figure S63** High resolution mass spectra of **Di-DTP**.

**[Ph-DTP-Me^+^][I^-^]**

**Figure S64** ^1^H NMR spectra of **[Ph-DTP-Me^+^][I^-^]** in CDCl_3_.

**Figure S65** ^13^C NMR spectra of **[Ph-DTP-Me^+^][I^-^]** in CDCl_3_.

**Figure S66** IR spectra of **[Ph-DTP-Me^+^][I^-^]**.

**Figure S67** High resolution mass spectra (ESI+) of **[Ph-DTP-Me^+^][I^-^]**.

**Figure S68** High resolution mass spectra (ESI-) of **[Ph-DTP-Me^+^][I^-^]**.

**[Ph-ACR-Me^+^][I^-^]**

**Figure S69** ^1^H NMR spectra of **[Ph-ACR-Me^+^][I^-^]** in CDCl_3_.

**Figure S70** ^1^C NMR spectra of **[Ph-ACR-Me^+^][I^-^]** in CDCl_3_.

**Figure S71** IR spectra of **[Ph-ACR-Me^+^][I^-^]**.

**Figure S72** High resolution mass spectra (ESI+) of **[Ph-ACR-Me^+^][I^-^]**.

**Figure S73** High resolution mass spectra (ESI-) spectra of **[Ph-ACR-Me^+^][I^-^]**.

**8.	References**

[1] C. M. S. Jones, A. Gakamsky and J. Marques-Hueso, “The upconversion quantum yield (UCQY): a review to standardize the measurement methodology, improve comparability, and define efficiency standards” *Science and Technology of Advanced Materials* **22**, (2021): 810.
[2] G. M. Sheldrick, “A short history of ShelX” *Acta Crystallographica Section A* **A64**, (2008): 339.
[3] O. V. Dolomanov, L. J. Bourhis, R. J. Gildea, J. A. K. Howard and H. Puschmann, “OLEX2 : a complete structure solution, refinement and analysis program” *Journal of Applied Crystallography* **42**, (2009): 339.
[4] L. J. Bourhis, O. V. Dolomanov, R. J. Gildea, J. A. K. Howard and H. Puschmann, “The Anatomy of a Comprehensive Constrained, Restrained, Refinement Program for the Modern Computing Environment - Olex2 Disected” *Acta Crystallographica Section A* **A71**, (2015): 59.
[5] D. Taylor, L. Amicosante, L. M. Luze, M. R. S. McCoustra, L. McMahon, S. J. Dalgarno and F. Vilela, “A Six-Week Student-Led Project Designed to Provide Insight into Modern Photochemistry Research” *Journal of Chemical Education* **102**, (2025): 1511.
[6] D. Taylor, J. M. Tobin, L. Amicosante, A. W. Prentice, M. J. Paterson, S. J. Dalgarno, N. B. McKeown and F. Vilela, “Immobilisation of benzo[*c*][1,2,5]thiadiazole (BTZ) within polymers of intrinsic microporosity (PIMs) for use in flow photochemistry” *Journal of Materials Chemistry A* **12***,* (2024): 10932.
[7] J. M. Barker, P. R. Huddleston and M. L. Wood, “An Easy Synthesis of 3-Amino- and 3-Nitrothiophene” *Synthetic Communications* **25**, (1995): 3729.
[8] https://www.sigmaaldrich.com/deepweb/assets/sigmaaldrich/marketing/global/documents/614/456/labbasics_pg144.pdf, accessed October 2025.
[9] Gaussian 16, Revision A.03, M. J. Frisch, G. W. Trucks, H. B. Schlegel, G. E. Scuseria, M. A. Robb, J. R. Cheeseman, G. Scalmani, V. Barone, G. A. Petersson, H. Nakatsuji, X. Li, M. Caricato, A. V. Marenich, J. Bloino, B. G. Janesko, R. Gomperts, B. Mennucci, H. P. Hratchian, J. V. Ortiz, A. F. Izmaylov, J. L. Sonnenberg, D. Williams-Young, F. Ding, F. Lipparini, F. Egidi, J. Goings, B. Peng, A. Petrone, T. Henderson, D. Ranasinghe, V. G. Zakrzewski, J. Gao, N. Rega, G. Zheng, W. Liang, M. Hada, M. Ehara, K. Toyota, R. Fukuda, J. Hasegawa, M. Ishida, T. Nakajima, Y. Honda, O. Kitao, H. Nakai, T. Vreven, K. Throssell, J. A. Montgomery, Jr., J. E. Peralta, F. Ogliaro, M. J. Bearpark, J. J. Heyd, E. N. Brothers, K. N. Kudin, V. N. Staroverov, T. A. Keith, R. Kobayashi, J. Normand, K. Raghavachari, A. P. Rendell, J. C. Burant, S. S. Iyengar, J. Tomasi, M. Cossi, J. M. Millam, M. Klene, C. Adamo, R. Cammi, J. W. Ochterski, R. L. Martin, K. Morokuma, O. Farkas, J. B. Foresman, and D. J. Fox, Gaussian, Inc., Wallingford CT, 2016.
[10] M. Bursch, J.-M. Mewes, A. Hansen and S. Grimme, “Best-Practice DFT Protocols for Basic Molecular Computational Chemistry” *Angewandte Chemie International Edition* **61**, (2022): e202205735.
